# Association Between Diet Quality and Prevalence of Obesity, Dyslipidemia, and Insulin Resistance Among Filipino Immigrant Women in Korea: The Filipino Women's Diet and Health Study

**DOI:** 10.3389/fpubh.2021.647661

**Published:** 2021-07-01

**Authors:** Hee Sun Kim, Heejin Lee, Sherlyn Mae P. Provido, Minji Kang, Grace H. Chung, Sangmo Hong, Sung Hoon Yu, Chang Beom Lee, Jung Eun Lee

**Affiliations:** ^1^Department of Food and Nutrition, College of Human Ecology, Seoul National University, Seoul, South Korea; ^2^Research Institute of Human Ecology, Seoul National University, Seoul, South Korea; ^3^BK21 FOUR Education and Research Team for Sustainable Food & Nutrition, Seoul, South Korea; ^4^Department of Child Development and Family Studies, College of Human Ecology, Seoul National University, Seoul, South Korea; ^5^Division of Endocrinology and Metabolism, Department of Internal Medicine, Hanyang University Guri Hospital, Hanyang University College of Medicine, Guri, South Korea

**Keywords:** diet quality, dietary diversity, data derived inflammatory index, obesity, dyslipidemia, insulin resistance, filipino immigrants, cross-sectional study

## Abstract

**Objectives:** Diet quality may be a key modifiable factor for the prevention of non-communicable disease. We aimed to investigate the association between diet quality and prevalence of obesity, dyslipidemia, and insulin resistance among Filipino immigrant women in Korea.

**Methods:** A total of 413 participants from the 2014–2016 baseline population of the Filipino Women's Diet and Health Study (FiLWHEL) were examined. Individual dietary intakes were evaluated through 24-h recalls and then converted into two dietary quality assessments: Minimum Dietary Diversity for Women (MDD-W) developed by the Food and Agriculture Organization (FAO) and the Data Derived Inflammation Index (DDII) originally developed by our group. Fasting blood levels of triglycerides, high-density lipoprotein cholesterols, glucose, and insulin were measured. We used logistic regression models for odds ratios (ORs) with 95% confidence intervals (CIs).

**Results:** We found a statistically significant association between MDD-W scores and decreased prevalence of abdominal obesity; ORs (95% CIs) of the 3rd vs. 1st tertiles were 0.58 (0.36–0.94; *p* for trend = 0.029). Increased DDII was associated with elevated prevalence of dyslipidemia and insulin resistance; ORs (95% CIs) of the 5th vs. 1–3rd quintiles were 6.44 (2.56–16.20) for triglycerides (TG), 3.90 (1.92–7.90) for low-density lipoprotein (LDL) cholesterol, 3.36 (1.81–6.24) for total cholesterol (TC), 6.25 (2.53–15.41) for abnormal TG/HDL ratios, 3.59 (1.96–6.59) for HbA1c, 2.61 (1.11–6.17) for fasting blood glucose levels, 9.67 (4.16–22.48) for insulin levels, and 9.73 (4.46–21.25) for homeostasis model assessment of insulin resistance (HOMA-IR) (*p* for trend <0.001 for all, except 0.033 for fasting blood glucose).

**Conclusions:** Greater dietary diversity was inversely associated with the prevalence of abdominal obesity in Filipino immigrant women. Proinflammatory scores based on diet and lifestyle factors were associated with an increased prevalence of dyslipidemia and insulin resistance. Further, epidemiological studies on the relationship between dietary acculturation and chronic disease are warranted.

## Introduction

With the rise of non-communicable disease—mainly obesity, type 2 diabetes mellitus (T2DM), and cardiovascular disease (CVD) (1–3)—in both developed and developing countries, significant changes were made in the public health priorities for their prevention. Of note, modern dietary guidelines not only aim to prevent nutrient deficiencies but also aim to prevent chronic disease. Conventional “nutrient-based” guidelines showed considerable limitations when extending to chronic disease prevention; thus, the translation of “nutrient-based” into “food-based” guidelines has been accentuated recently (4–6). Indeed, the growing burden of non-communicable diseases has led to demand for a holistic approach to food consumption that can manage the double burden of malnutrition, under-, and over-nutrition. “Food-based” guidelines attempt to reflect the synergistic contributions of dietary patterns, including food composition, preparation methods, and underlying social interactions. To ensure the compliance and effectiveness of established guidelines, quantitative measurements such as diet quality scores have been developed using both empirical and a priori methods such as Mediterranean diet score, Healthy Eating Index (HEI), and Alternative Healthy Eating Index (AHEI) (7, 8).

Several epidemiological studies have examined the association between diet quality and morbidity and mortality from non-communicable disease, where heterogeneous outcomes were found, including significant positive (9–11), inverse (12, 13), or no associations (14). The Nurses' Health Study comprising 80,029 participants reported a statistically significant association between higher AHEI and a lower risk of T2DM (9). However, no significant association was found in a meta-analysis examining the association between dietary diversity score (DDS) and obesity (15). Dietary factors associated with inflammation status have been highlighted, along with the recent development of dietary index predicting inflammation levels (16–20). Chronic inflammation status is recognized as an underlying pathophysiological mechanism linking multiple chronic diseases, including atherosclerosis (21), T2DM (22), CVD (23), and several cancers (24). The heterogeneous results of the previous literature suggest the need for a population-specific diet quality assessment tool regarding the association between non-communicable diseases.

In our previous study, we developed Data Derived Inflammatory Index (DDII) based on high-sensitive C-reactive protein (hsCRP) levels (20). It is a Korean-specific index that reflects both diet and lifestyle factors with major attributes to hsCRP levels. Food and Agriculture Organization (FAO) of the United Nations developed the Minimum Dietary Diversity for Women (MDD-W), a food group-based dichotomous scoring system, designed to assess the micronutrient adequacy of the women of reproductive age in developing countries (25). Several epidemiologic studies found the association between MDD-W and multiple chronic diseases (26–28).

Of note, the highest prevalence of obesity was reported among Filipino women from the immigrant study in Korea (29). Another immigrant study in the United States found that the prevalence of obesity in Filipinos tripled between 1992 and 2011, which was the highest among Asian immigrants (30). Our group found an inverse association between obesity prevalence and higher DDS based on 11 food groups (31). Abdominal obesity is a distinct phenotype of the obesity pandemic in developing countries, strongly associated with metabolic comorbidities (32–34). In addition, Asians may have a higher body fat mass at a given value of BMI compared with Caucasians (35). Based on this rationale, international institutions have proposed population-specific cutoff points for the diagnosis of obesity and metabolic syndrome (19, 36, 37). In tandem, population-specific public health intervention strategies, including dietary guidelines to prevent non-communicable disease, should be enforced concerning the metabolic differences.

Given that Filipino immigrant women are susceptible to lower dietary diversity and metabolic diseases, we aimed to examine whether MDD-W, a developing country-specific score, and DDII, a Korean-specific score, were associated with obesity and other metabolic comorbidities among Filipino immigrant women in Korea. We examined the associations between MDD-W and DDII and indicators of obesity, dyslipidemia, and insulin resistance in the Filipino Women's Diet and Health Study (FiLWHEL).

## Methods

### The Filipino Women's Diet and Health Study

This was a cross-sectional study of 504 baseline population in the FiLWHEL, an ongoing prospective cohort study of married immigrant Filipino women in the Republic of Korea. Between March 2014 and April 2016, baseline participants were recruited from various regions in Korea, including Seoul, Incheon, Daejeon, and the rural areas of Gyeonggi and Chungcheong provinces. The prerequisites for the enrollment were the following: (1) aged 19 years or over, and (2) ever having been married to a Korean man with Korean citizenship, while the participant may be married, widowed, or divorced at the point of enrollment. A total of 434 participants were examined after the exclusion of 70 participants due to the following reasons: (1) participants with incomplete data of 24-h dietary records (*n* = 7), anthropometric measurements (*n* = 8), or blood lipid profile (*n* = 11); (2) participants who were currently pregnant (*n* = 19) or breastfeeding (*n* = 56); and (3) participants with an implausible range of energy intake (>±3 SDs) (*n* = 13).

The study design attempted to investigate health, social, and dietary factors in Filipino immigrant women in Korean society. A detailed description of the FiLWHEL study has been mentioned elsewhere (38). Participants' data were collected by health-related and socioeconomic questionnaires, anthropometric measurements, and biospecimen collection. The respondents completed questionnaires either on-site or *via* phone interview. Filipino volunteers were involved in the data collection to clarify the communication through Tagalog. Responses were immediately reviewed and verified before the participants were dismissed. Written informed consents were collected from all study participants. This study was approved by the Institutional Review Board of Sookmyung Women's University (SMWU-1311-BR-012).

### Dietary Assessment and Computation of Diet Quality Scores

A single-day 24-h recall was administered at the baseline, with standard portion sizes and units provided using food miniatures, photographs, household measures, weight, and volume. The collected dietary data were computed by CAN-pro 4.0 (Computer Aided Analysis Program 4.0 for professionals, Korean Society of Nutrition, Seoul, South Korea). Foods absent from the Korean database were supplemented from foreign resources: food composition tables of the Food and Nutrition Research Institute of the Philippines (39), Korean Rural Development Administration (40), and the U.S. Department of Agriculture (16).

The MDD-W was developed by FAO through the Women's Dietary Diversity Project (WDDP) as a proxy indicator for micronutrient adequacy targeting the women of reproductive age (WRA) in developing countries (25). MDD-W is composed of 10 food groups, where 1 point is assigned for consuming ≥15 g (1 serving) of each food group, and 0 point is assigned for consuming less. The selected food groups are as follows: (1) grains, white roots, and tubers; (2) pulses (beans, peas, and lentils); (3) nuts and seeds; (4) dairy; (5) meat, poultry, and fish; (6) eggs; (7) dark green leafy vegetables; (8) other vitamin A-rich fruits and vegetables; (9) other vegetables; and (10) other fruits. The total score ranges from 0 to 10 points, where 5 points are used as cutoff value to achieve the minimum micronutrient adequacy. In this study, energy-adjusted MDD-W score was calculated per 1,000 kcal of food consumption considering the effect of total energy intake. We computed the energy-adjusted MDD-W score according to the original criteria. The DDII based on hsCRP levels was developed by our group (20). DDII is a Korean-specific predicted hsCRP score derived from dietary, lifestyle, and health-related data of 23,330 participants in the Health Examinee Study (HEXA). The HEXA study is the largest subset of the Korean Genome and Epidemiology Study (KoGES), a cohort study investigating the epidemiologic and genomic factors associated with chronic disease in the Korean population (41). A combined and sex-specific index is available, and the predicted hsCRP score is calculated as a sum of multiplying beta coefficients by corresponding factors. We computed the DDII score using women-specific criteria: (1) age, (2) BMI, (3) smoking status (never, past, current), (4) menopausal status (premenopausal, perimenopausal, postmenopausal), (5) education level (elementary school or below, middle school, high school, university or above), (6) beef, (7) processed fish, (8) soup and stew with soybean paste/soybean paste, (9) sweet bread, and (10) fish. A higher DDII score indicates increased hsCRP levels and more proinflammatory status.

### Anthropometric Measurements, Blood Sampling, and Other Variables

Anthropometric data including height, weight, and waist circumference were collected on-site. BMI was calculated as the ratio of weight (kg) to the square of height (m^2^) measurements. Blood samples were collected after a minimum of 8-h fasting. After a professional phlebotomist drew blood under the supervision of a medical doctor, the sample was immediately centrifuged and refrigerated until the analysis within 24 h. The serum triglycerides (TG), total cholesterol (TC), fasting blood glucose (FBG), and high-density lipoprotein (HDL) cholesterol levels were analyzed *via* the Cobas 8000 C702-I (Roche Diagnostics, Basel, Switzerland). Glycated hemoglobin (HbA1c) levels were measured using the Tosoh G8 by high-performance liquid chromatography (HPLC) analyzer (Tosoh Bioscience, Tokyo, Japan). The intra-assay coefficients of variation (CV) for each biomarker were 1.49–2.99% for total cholesterol, 1.48–2.33% for blood triglycerides, 0.98–2.11% for high-density lipoprotein cholesterols, and 1.21–2.79% for fasting blood glucose. Serum low-density lipoprotein (LDL) cholesterol levels were calculated indirectly through the Friedewald formula as follows: LDL cholesterol = TC – HDL cholesterol – (TG/5) (42). We used the original homeostatic model assessment (HOMA) model of Matthews et al. (43) to calculate homeostasis model assessment of insulin resistance (HOMA-IR) estimates from fasting blood glucose and insulin measurements; HOMA-IR = fasting insulin (μIU/ml) × fasting glucose (mmol/ml)/22.5.

For the obesity classification, two determinants were used with cutoff values according to a joint effort of the regional office for the Western Pacific of the World Health Organization, the International Association for the Study of Obesity, and the International Obesity Task Force (36): waist circumference ≥80 cm and BMI ≥25 kg/m^2^. We used four lipid parameters of dyslipidemia from the 2018 Korean Guidelines for the Management of Dyslipidemia (44), and levels above the normal or optimal range were considered as cutoff values. The four parameters were (1) total cholesterol ≥200 mg/dl, (2) LDL-cholesterol ≥130 mg/dl, (3) triglycerides ≥150 mg/dl, or (4) HDL-cholesterol <40 mg/dl. The prediabetes cutoffs from the 2018 recommendations of the American Diabetes Association (ADA) were used for the analysis of insulin resistance (45); HbA1c ≥5.7% or FBG ≥100 mg/dl. Other indicators related to dyslipidemia and insulin resistances such as TG/HDL ratios and HOMA-IR were also examined based on the cutoff from the recent studies (5, 43, 46, 47).

Information on age (years), education level (elementary school, high school, associate/vocational, college/university, graduate school), employment status (yes, no), current smoking status (yes, no), smoking cohabitants (yes, no), sleep behaviors, physical activity (vigorous, moderate, walking, sitting), parity, breastfeeding length (months), menopausal status, place of residence (Seoul, Incheon, Daejeon, Kyeonggi, Cheongnam), migrant year, and dietary behavior changes during the past year (yes, no) was obtained from the structured questionnaires. Energy (kcal), carbohydrate (g), protein (g), and fat (g) intakes were calculated from the 24-h recall data. Overall quality of life (QOL) was assessed using the World Health Organization Quality of Life-BREF (WHOQOL-BREF). Coffee intake per day was calculated by summing up all the coffee types after multiplying serving sizes by consumption frequencies during the previous year. Past-year alcohol consumption was estimated by collective intake of ethanol intake from soju, beer, liquor, wine, rice wine, and refined rice wine during the past year. The ethanol intake in grams per day was calculated using the percentage of alcohol in the liquor, the mass of ethanol in 1 L of liquor, and the serving size. The applied formula is as follows: alcohol (g) = alcoholic content of liquor/100 × (0.7893) × serving size (cc).

### Statistical Analyses

For the descriptive analysis, the participants were ranked into tertiles of MDD-W and quintiles of DDII scores. We used the chi-square test for the categorical variables and ANOVA for the continuous variables to compare across each category by means with SDs or frequencies. Multiple logistic regression model was used to determine odds ratios (ORs) with 95% confidence intervals (CIs) for the association between diet quality scores and obesity and components of dyslipidemia. For this analysis, MDD-W was categorized into tertiles, while DDII was categorized into quintiles and then re-categorized into three groups by assembling the three lowest categories to assure a substantial number of cases. For each diet quality score, model 1 was adjusted for age (years, continuous) and energy intake (kcal, continuous). For the analysis of MDD-W and obesity, model 2 was additionally adjusted for dietary behavior changes (yes, no), employment status (yes, no), smoking cohabitant (yes, no), and region (urban, rural). Model 2 of the dyslipidemia and insulin resistance analyses and model 3 of the abdominal obesity analysis were additionally adjusted for BMI (<20, 20– <23, 23– <25, ≥25 kg/m^2^). For the analysis of DDII, model 2 was additionally adjusted for education level (high school or less, associate/vocational, college or above), sleep hours (<5, 5–6, 7–8, >8 h), vigorous activity (yes, no), breastfeeding length (months, continuous), dietary behavior change (yes, no), and alcohol intake (yes, no). Model 3 was additionally adjusted for BMI (<20, 20– <23, 23– <25, ≥25 kg/m^2^). Multiple logistic regression was used for the stratified analysis to assess the possible effect modification factor of the association between diet quality score and outcome variables. Age (<35, ≥35 years), BMI (<25, ≥25 kg/m^2^), and length of residence in Korea (≤ 8, >8 years) were examined. To evaluate whether DDII predicted hsCRP levels in this population, a general linear model (GLM) analysis for least-squares means (LS-means) with 95% CIs was used to calculate the mean levels of hsCRP according to the quintiles of DDII. To improve the normality, logarithmical transformation was applied to hsCRP levels.

For the sensitivity analyses, MDD-W was examined as quartiles and continuous variable, and DDII was examined as quintiles, using the logistic regression model. BMI was also analyzed as a continuous variable. In addition, both diet quality scores were analyzed using GLM analysis for LS-means with 95% CIs, to calculate the mean levels of each outcome. Logarithmical transformation was applied to obesity, dyslipidemia, and insulin resistance measurements to improve the normal distribution. We also examined the association particularly among the WRA for MDD-W analysis, and actual hsCRP levels were assessed using the logistic regression model. All statistical analyses were conducted *via* SAS version 9.4 (SAS Institute Inc., Cary, NC, United States); all tests were two-sided, and the significance level was set at *P* < 0.05.

## Results

A total of 413 participants from the baseline population of the FiLWHEL study were included in the analysis, with variation regarding the major outcomes; 413 participants were included for general obesity (no. of cases = 124), 411 participants were included for abdominal obesity (no. of cases = 177), and 405 participants were included in the dyslipidemia and insulin resistance analyses (no. of cases = 39 for TG, 28 for HDL, 76 for LDL, 109 for TC, 43 for TG/HDL, 141 for HbA1c, 43 for FBG, 57 for insulin levels, and 65 for HOMA-IR). The baseline characteristics according to each diet quality score are outlined in Tables 1, 2. MDD-W scores did not vary across DDII scores, nor did DDII scores according to MDD-W scores. The individuals with greater MDD-W scores tended to have higher % energy from carbohydrate intake but lower % energy from fat intake compared to those with lower MDD-W scores. The participants with higher DDII scores tended to be older, had higher BMI, higher % energy from carbohydrate intake but lower % energy from fat intake, and higher fasting blood glucose levels compared to those with lower DDII scores. Engagement in vigorous activity was higher among individuals with high MDD-W scores than among those with low scores. The proportions of current alcohol consumers were the lowest in the top categories of MDD-W and DDII scores, while the proportions of participants who lived more than 8 years in Korea were the highest in the top categories of both scores. The mean (±SD) score was 4.79 (±1.53) for MDD-W and 2.33 (±0.36) for DDII.

**Table 1:** Baseline characteristics according to the tertiles of MDD-W in the FiLWHEL study.

	**MDD-W (*****n*** **=** **413)**		
	**≤4**	**5**	**≥6**
	**Mean (±SD)**		
Number of participants	179	95	139
DDII^†^	2.34 (0.37)	2.38 (0.37)	2.32 (0.32)
Age, years	34.45 (8.06)	35.27 (8.17)	36.26 (7.69)
Body mass index, kg/m^2^	23.71 (4.18)	24.17 (3.99)	23.22 (3.32)
Energy intake, kcal/day	1727.32 (702.23)	1712.81 (621.34)	1787.20 (560.68)
Carbohydrate intake, g/day	239.03 (105.19)	235.03 (96.54)	262.41 (90.89)
Protein intake, g/day	66.03 (35.08)	73.28 (29.31)	72.62 (28.86)
Fat intake, g/day	54.72 (32.28)	52.73 (31.17)	50.69 (25.96)
%Carbohydrate intake	56.55 (12.48)	55.43 (10.89)	58.71 (9.88)
%Protein intake	15.32 (4.85)	17.35 (4.32)	16.17 (4.00)
%Fat intake	28.12 (10.24)	27.22 (9.60)	25.12 (8.67)
Waist circumferences, cm	79.78 (9.72)	80.72 (9.89)	78.27 (8.57)
Triglycerides, mg/dl	86.10 (42.07)	87.15 (45.83)	92.04 (54.50)
High-density lipoprotein cholesterol, mg/dl	58.10 (13.46)	58.84 (12.61)	57.48 (14.96)
Low-density lipoprotein cholesterol, mg/dl	101.95 (32.02)	102.57 (29.57)	107.50 (29.70)
Total cholesterol, mg/dl	177.27 (34.67)	178.84 (34.13)	183.39 (35.34)
TG/HDL	1.64 (1.15)	1.61 (1.16)	1.87 (1.73)
HbA1c, %	5.50 (0.76)	5.55 (0.87)	5.48 (0.53)
Fasting blood glucose, mg/dl	88.47 (9.61)	88.79 (13.74)	88.52 (13.14)
Insulin, mg/dl	10.08 (10.75)	9.24 (6.95)	10.08 (9.01)
HOMA-IR	2.30 (2.89)	2.10 (1.98)	2.31 (2.40)
Breastfeeding length, month	8.17 (13.13)	8.21 (11.98)	10.14 (13.27)
Quality of life	57.70 (7.39)	58.06 (8.02)	58.68 (6.87)
	***n*** **(%)**		
**Education level**			
Associate vocational or less	81 (45.25)s	37 (38.95)	57 (41.01)
College graduate or over	98 (54.75)	58 (61.05)	82 (58.99)
**Employment status**			
No	106 (59.22)	52 (54.74)	69 (49.64)
Yes	73 (40.78)	43 (45.26)	70 (50.36)
Vigorous activity^*^			
No	150 (83.80)	80 (84.21)	107 (76.98)
Yes	29 (16.20)	15 (15.79)	32 (23.02)
**Alcohol intake**			
No	62 (34.64)	32 (33.68)	54 (38.85)
Yes	117 (65.36)	63 (66.32)	85 (61.15)
**Smoking cohabitant**			
No	160 (89.39)	87 (91.58)	133 (95.68)
Yes	19 (10.61)	8 (8.42)	6 (4.32)
Length of residence, years^**^			
≤ 8	106 (59.22)	51 (53.68)	73 (52.52)
>8	73 (40.78)	44 (46.32)	66 (47.48)
**Region**			
Urban	76 (42.46)	32 (33.68)	48 (34.53)
Rural	103 (57.54)	63 (66.32)	91 (65.47)

*MDD-W, Minimum Dietary Diversity for Women; FiLWHEL, Filipino Women's Diet and Health Study*.

† *Data Derived Inflammatory Index*.

* *Participating in a vigorous activity each week*.

** *Years of residence in the Republic of Korea*.

**Table 2:** Baseline characteristics according to the quintiles of DDII in the FiLWHEL study.

	**DDII (*****n*** **=** **405)**				
	**Q1**	**Q2**	**Q3**	**Q4**	**Q5**
	**Mean (±SD)**				
Total population number	81	81	81	81	81
MDD-W^†^	4.88 (1.58)	4.48 (1.54)	4.91 (1.57)	4.78 (1.41)	4.75 (1.49)
Age, years	29.57 (5.49)	32.78 (5.79)	34.72 (7.06)	37.62 (7.72)	40.56 (8.21)
Body mass index, kg/m^2^	19.51 (2.73)	21.55 (1.66)	23.49 (1.72)	24.49 (1.94)	28.86 (3.68)
Energy intake, kcal/day	1749.34 (625.71)	1718.85 (620.09)	1698.68 (601.85)	1760.24 (739.36)	1793.88 (600.14)
Carbohydrate intake, g/day	244.52 (91.09)	236.10 (96.36)	229.81 (98.17)	256 (108.70)	261.66 (99.13)
Protein intake, g/day	66.51 (29.07)	69.20 (34.01)	72.45 (30.46)	70.67 (37.15)	71.60 (32.27)
Fat intake, g/day	55.71 (32.59)	54.31 (30.22)	53.35 (29.21)	50.98 (31.32)	50.43 (26.63)
%Carbohydrate intake	56.83 (10.93)	55.76 (11.81)	54.46 (12.98)	58.91 (10.65)	58.88 (10.40)
%Protein intake	15.28 (4.23)	16.15 (4.82)	17.21 (4.35)	15.84 (4.71)	16.04 (4.97)
%Fat intake	27.88 (9.57)	28.09 (10.03)	28.33 (10.74)	25.25 (8.61)	25.08 (8.97)
Waist circumferences, cm	70.91 (6.91)	75.52 (5.33)	78.29 (5.55)	81.90 (5.55)	90.44 (9.02)
Triglycerides, mg/dl	68.91 (30.57)	81.57 (39.98)	82.81 (33.75)	94.72 (54.21)	114.28 (59.06)
High-density lipoprotein cholesterol, mg/dl	59.59 (13.34)	60.90 (13.56)	55.52 (13.73)	59.60 (14.66)	54.36 (12.50)
Low-density lipoprotein cholesterol, mg/dl	91.14 (24.89)	99.39 (29.44)	103.67 (29.77)	108.22 (29.66)	116.48 (33.63)
Total cholesterol, mg/dl	164.52 (28.84)	176.60 (33.65)	175.75 (32.53)	186.77 (33.52)	193.69 (37.81)
TG/HDL	1.24 (0.68)	1.46 (1.02)	1.65 (0.94)	1.85 (1.80)	2.38 (1.77)
HbA1c, %	5.35 (0.46)	5.30 (0.31)	5.43 (0.41)	5.49 (0.37)	5.95 (1.31)
Fasting blood glucose, mg/dl	84.47 (7.06)	87.09 (8.17)	88.20 (12.21)	89.42 (14.01)	93.53 (14.03)
Insulin, mg/dl	6.32 (3.45)	7.85 (4.84)	9.41 (6.01)	11.49 (11.76)	14.56 (14.05)
HOMA-IR	1.33 (0.76)	1.71 (1.13)	2.07 (1.40)	2.73 (3.66)	3.47 (3.54)
Breastfeeding length, month	8.77 (10.47)	7.71 (10.22)	8.73 (11.24)	10.57 (17.86)	7.96 (12.36)
Quality of life	58.88 (6.48)	58.44 (8.17)	57.37 (7.29)	57.88 (6.72)	57.26 (7.72)
	***n*** **(%)**				
**Education level**					
Associate vocational or less	52 (64.20)	26 (32.10)	41 (50.62)	24 (29.63)	30 (37.04)
College graduate or over	29 (35.80)	55 (67.90)	40 (49.38)	57 (70.37)	51 (62.96)
**Employment status**					
No	52 (64.20)	52 (64.20)	49 (60.49)	36 (44.44)	37 (45.68)
Yes	29 (35.80)	29 (35.80)	32 (39.51)	45 (55.56)	44 (54.32)
Vigorous activity^*^					
No	65 (80.25)	68 (83.95)	62 (76.54)	69 (85.19)	66 (81.48)
Yes	16 (19.75)	13 (16.05)	19 (23.46)	12 (14.81)	15 (18.52)
**Alcohol intake**					
No	28 (34.57)	22 (27.16)	32 (39.51)	28 (34.57)	34 (41.98)
Yes	53 (65.43)	59 (72.84)	49 (60.49)	53 (65.43)	47 (58.02)
**Smoking cohabitant**					
No	78 (96.30)	71 (87.65)	75 (92.59)	74 (91.36)	74 (91.36)
Yes	3 (3.70)	10 (12.35)	6 (7.41)	7 (8.64)	7 (8.64)
Length of residence, years^**^					
≤ 8	63 (77.78)	56 (69.14)	50 (61.73)	36 (44.44)	25 (30.86)
>8	18 (22.22)	25 (30.86)	31 (38.27)	45 (55.56)	56 (69.14)
**Region**					
Urban	36 (44.44)	25 (30.86)	31 (38.27)	37 (45.68)	28 (34.57)
Rural	45 (55.56)	56 (69.14)	50 (61.73)	44 (54.32)	53 (65.43)

*DDII, Data Derived Inflammatory Index; FiLWHEL, Filipino Women's Diet and Health Study*.

† *Minimum Dietary Diversity for Women*.

* *Participating in a vigorous activity each week*.

** *Years of residence in the Republic of Korea*.

We observed a statistically significant linear trend of increased hsCRP levels according to the quintiles of DDII in the age- and energy-adjusted model; LS-means (95% CIs) were 0.79 (0.61–1.02) for quintile 1, 0.96 (0.76–1.22) for quintile 2, 1.02 (0.82–1.28) for quintile 3, 1.14 (0.90–1.45) for quintile 4, and 1.65 (1.30–2.09) for quintile 5 (*p* for trend < .001) (Supplementary Table 1).

The associations between the tertiles of MDD-W and obesity, dyslipidemia, and insulin resistance markers are presented in Table 3. We observed a statistically significant association between wider dietary variety and lower prevalence of central obesity, while higher prevalence of dyslipidemia. The multivariable adjusted ORs (95% CIs) of the 3rd vs. the 1st tertiles of MDD-W were, 0.58 (0.36–0.94) for central obesity, 2.43 (1.11–5.36) for hypertriglyceridemia, 2.02 (1.17–3.47) for hypercholesterolemia, and 3.37 (1.53–7.43) for abnormal TG/HDL ratio (*p* for trend: 0.029, 0.029, 0.013, and 0.003, respectively). We also found a suggestive inverse trend between MDD-W and lower prevalence of general obesity but an association or trend was not statistically significant. No statistically significant association was found between MDD-W scores and biomarkers of insulin resistance (HbA1c, FBG, insulin, and HOMA-IR).

**Table 3:** Odds ratios (ORs) and 95% confidence intervals (CIs) of BMI, waist circumferences, triglycerides, HDL-C, LDL-C, total cholesterol, TG/HDL, HbA1c, fasting blood glucose, insulin, and HOMA-IR, according to the MDD-W scoring in the FiLWHEL study.

	**ORs (95% CIs) per 1 score increment**	**ORs (95% CIs) according to the tertiles of MDD-W**			
		**≤4**	**5**	**≥6**	***p-trend***
**Body mass index (≥25 kg/m**^**2**^**)**					
Case/total number		(57/179)	(32/95)	(35/139)	
Model 1^*^	0.93 (0.81–1.06)	1	1.06 (0.62–1.81)	0.67 (0.41–1.11)	0.138
Model 2^**^	0.92 (0.80–1.06)	1	1.05 (0.61–1.80)	1.05 (0.61–1.08)	0.108
**Waist circumferences (≥80 cm)**					
Case/total number		(82/176)	(43/96)	(52/139)	
Model 1^*^	0.90 (0.79–1.03)	1	0.88 (0.53–1.47)	0.60 (0.37–0.96)	0.036
Model 2^**^	0.90 (0.79–1.03)	1	0.89 (0.53–1.50)	0.58 (0.36–0.94)	0.029
Model 3^†^	0.89 (0.74–1.08)	1	0.70 (0.33–1.49)	0.56 (0.28–1.12)	0.097
**Triglycerides (≥150 mg/dl)**					
Case/total number		(13/177)	(7/96)	(19/132)	
Model 1^*^	1.28 (1.02–1.61)	1	0.96 (0.37–2.52)	2.04 (0.96–4.32)	0.062
Model 2^†^	1.35 (1.06–1.71)	1	0.88 (0.32–2.38)	2.43 (1.11–5.36)	0.029
**High-density lipoprotein cholesterol (<40 mg/dl)**					
Case/total number		(11/177)	(3/96)	(14/132)	
Model 1^*^	1.15 (0.89–1.49)	1	0.49 (0.13–1.80)	1.86 (0.81–4.27)	0.150
Model 2^†^	1.19 (0.90–1.56)	1	0.45 (0.12–1.68)	2.19 (0.92–5.22)	0.087
**Low-density lipoprotein cholesterol (≥130 mg/dl)**					
Case/total number		(32/177)	(16/96)	(28/132)	
Model 1^*^	1.01 (0.85–1.19)	1	0.87 (0.45–1.71)	1.15 (0.64–2.04)	0.668
Model 2^†^	1.05 (0.88–1.25)	1	0.88 (0.43–1.80)	1.38 (0.75–2.56)	0.325
**Total cholesterol (≥200 mg/dl)**					
Case/total number		(40/177)	(23/96)	(46/132)	
Model 1^*^	1.18 (1.01–1.37)	1	1.05 (0.57–1.91)	1.75 (1.04–2.92)	0.036
Model 2^†^	1.22 (1.04–1.43)	1	1.04 (0.55–1.96)	2.02 (1.17–3.47)	0.013
**TG/HDL (≥3)**					
Case/total number		(13/177)	(8/96)	(22/132)	
Model 1^*^	1.34 (1.07–1.67)	1	1.14 (0.45–2.86)	2.54 (1.22–5.29)	0.012
Model 2^†^	1.44 (1.14–1.84)	1	1.15 (0.44–3.02)	3.37 (1.53–7.43)	0.003
**HbA1c (≥5.7%)**					
Case/total number		(58/177)	(37/96)	(46/132)	
Model 1^*^	1.01 (0.88–1.16)	1	1.27 (0.74–2.18)	0.99 (0.60–1.63)	0.980
Model 2^†^	1.02 (0.88–1.18)	1	1.18 (0.67–2.07)	1.05 (0.63–1.76)	0.817
**Fasting blood glucose (≥100 mg/dl)**					
Case/total number		(19/177)	(11/96)	(13/132)	
Model 1^*^	0.95 (0.77–1.17)	1	1.02 (0.45–2.31)	0.81 (0.38–1.74)	0.602
Model 2^†^	0.95 (0.76–1.19)	1	1.01 (0.43–2.36)	0.83 (0.38–1.84)	0.662
**Insulin (≥15 mg/dl)**					
Case/total number		(23/177)	(13/96)	(21/132)	
Model 1^*^	1.07 (0.89–1.30)	1	1.09 (0.52–2.28)	1.38 (0.72–2.64)	0.342
Model 2^†^	1.14 (0.92–1.40)	1	1.15 (0.52–2.51)	1.70 (0.84–3.45)	0.148
**HOMA-IR (≥3.16)**					
Case/total number		(27/177)	(15/96)	(23/132)	
Model 1^*^	1.03 (0.87–1.23)	1	1.04 (0.52–2.07)	1.19 (0.64–2.19)	0.590
Model 2^†^	1.08 (0.89–1.31)	1	1.05 (0.51–2.18)	1.42 (0.73–2.74)	0.312

*FiLWHEL, Filipino Women's Diet and Health Study; MDD-W, Minimum Dietary Diversity for Women; HDL-C, High-Density Lipoprotein Cholesterol; LDL-C, Low-Density Lipoprotein Cholesterol; HbA1c, Hemoglobin A1c; BMI, Body mass index; TG/HDL, Triglycerides/High-density lipoprotein cholesterols; HOMA-IR, Homeostasis Model Assessment of Insulin Resistance*.

* *Model 1 adjusted for age (years, continuous) and energy intake (kcal, continuous)*.

** *Model 2 additionally adjusted for dietary behavior changes (yes, no), employment status (yes, no), smoking cohabitant (yes, no), and region (urban, rural)*.

† *Model 2 and Model 3 additionally adjusted for dietary behavior changes (yes, no), employment status (yes, no), smoking cohabitant (yes, no), region (urban, rural), and BMI (<20, 20- <23, 23- <25, ≥ 25 kg/m^2^)*.

We observed that the increased DDII scores were associated with an elevated prevalence of dyslipidemia and insulin resistance (Table 4). The multivariable adjusted ORs (95% CIs) of the 5th vs. 1–3rd quintiles of DDII were 6.44 (2.56–16.20) for triglycerides, 3.90 (1.92–7.90) for LDL cholesterol, 3.36 (1.81–6.24) for total cholesterol, 6.25 (2.53–15.41) for abnormal TG/HDL ratio, 3.59 (1.96–6.59) for HbA1c, 2.61 (1.11–6.17) for fasting blood glucose levels, 9.67 (4.16–22.48) for insulin levels, and 9.73 (4.46–21.25) for HOMA-IR (*p* for trend: <0.001 for all, except 0.033 for fasting blood glucose).

**Table 4:** Odds ratios (ORs) and 95% confidence intervals (CIs) of triglycerides, HDL-C, LDL-C, total cholesterols, TG/HDL, HbA1c, fasting blood glucose, insulin, HOMA-IR, and waist circumferences according to DDII in the FiLWHEL study.

	**ORs (95% CIs) according to three categories of DDII**			
	**Q1–3**	**Q4**	**Q5**	***p-trend***
**(*n* = 405)**	**247**	**77**	**81**	
**Triglycerides (≥150 mg/dl)**				
Case/total number	(12/247)	(8/77)	(19/81)	
Model 1^*^	1	2.21 (0.84–5.84)	5.78 (2.44–13.70)	<0.001
Model 2^**^	1	2.13 (0.77–5.90)	6.44 (2.56–16.20)	<0.001
Model 3^***^	1	2.41 (0.71–8.16)	7.67 (1.68–34.99)	0.009
**High-density lipoprotein cholesterol (<40 mg/dl)**				
Case/total number	(14/247)	(6/77)	(8/81)	
Model 1^*^	1	1.67 (0.59–4.71)	2.38 (0.86–6.53)	0.087
Model 2^**^	1	1.73 (0.60–4.99)	2.32 (0.83–6.52)	0.100
Model 3^***^	1	0.86 (0.25–3.00)	0.73 (0.17–3.14)	0.670
**Low-density lipoprotein cholesterol (≥130 mg/dl)**				
Case/total number	(27/247)	(18/77)	(31/81)	
Model 1^*^	1	2.16 (1.08–4.32)	4.14 (2.13–8.02)	<0.001
Model 2^**^	1	2.03 (0.98–4.19)	3.90 (1.92–7.90)	<0.001
Model 3^***^	1	1.74 (0.72–4.19)	2.43 (0.79–7.47)	0.118
**Total cholesterol (≥200 mg/dl)**				
Case/total number	(43/247)	(26/77)	(40/81)	
Model 1^*^	1	2.02 (1.10–3.68)	3.57 (1.96–6.49)	<0.001
Model 2^**^	1	1.95 (1.04–3.63)	3.36 (1.81–6.24)	<0.001
Model 3^***^	1	1.53 (0.73–3.18)	2.08 (0.81–5.36)	0.124
**TG/HDL (≥3)**				
Case/total number	(15/247)	(10/77)	(18/81)	
Model 1^*^	1	2.58 (1.07–6.24)	5.27 (2.29–12.11)	<0.001
Model 2^**^	1	2.86 (1.11–7.35)	6.25 (2.53–15.41)	<0.001
Model 3^***^	1	2.37 (0.77–7.25)	3.48 (0.89–13.58)	0.072
**HbA1c (≥5.7%)**				
Case/total number	(61/247)	(31/77)	(49/81)	
Model 1^*^	1	1.54 (0.87–2.74)	3.19 (1.79–5.70)	<0.001
Model 2^**^	1	1.86 (1.02–3.39)	3.59 (1.96–6.59)	<0.001
Model 3^***^	1	1.49 (0.74 – 3.00)	2.54 (1.03–6.24)	0.046
**Fasting blood glucose (≥100 mg/dl)**				
Case/total number	(15/247)	(9/77)	(19/81)	
Model 1^*^	1	1.35 (0.54–3.39)	2.71 (1.19–6.16)	0.020
Model 2^**^	1	1.14 (0.43–3.01)	2.61 (1.11–6.17)	0.033
Model 3^***^	1	0.79 (0.26–2.42)	1.30 (0.34–5.01)	0.741
**Insulin (≥15 mg/dl)**				
Case/total number	(21/247)	(16/77)	(20/81)	
Model 1^*^	1	5.17 (2.37–11.29)	8.70 (3.88–19.48)	<0.001
Model 2^**^	1	5.37 (2.38–12.09)	9.67 (4.16–22.48)	<0.001
Model 3^***^	1	2.85 (1.10–7.37)	3.60 (1.15–11.25)	0.025
**HOMA-IR (≥3.16)**				
Case/total number	(23/247)	(15/77)	(27/81)	
Model 1^*^	1	3.33 (1.57–7.06)	8.38 (4.01–17.52)	<0.001
Model 2^**^	1	3.37 (1.53–7.42)	9.73 (4.46–21.25)	<0.001
Model 3^***^	1	1.96 (0.78–4.93)	3.92 (1.31–11.71)	0.015
**(*****n*** **=** **411)**				
**Waist circumferences (≥80 cm)**				
Case/total number	(49/245)	(52/83)	(76/83)	
Model 1^*^	1	8.26 (4.49–15.17)	58.01 (23.13–145.52)	<0.001
Model 2^**^	1	9.01 (4.78–16.98)	69.04 (26.59–179.27)	<0.001
Model 3^***^	1	1.73 (0.78–3.86)	2.33 (0.68–8.05)	0.120

*FiLWHEL, Filipino Women's Diet and Health Study; DDII, Data Derived Inflammatory Index; HDL-C, High-Density Lipoprotein Cholesterol; LDL-C, Low-Density Lipoprotein Cholesterol; HbA1c, Hemoglobin A1c; BMI, Body mass index; TG/HDL, Triglycerides/High-density lipoprotein cholesterols; HOMA-IR, Homeostasis Model Assessment of Insulin Resistance*.

* *Model 1 adjusted for age (years, continuous) and energy intake (kcal, continuous)*.

** *Model 2 additionally adjusted for education level (high school or less, associate/vocational, college or above), sleep hours (<5, 5–6, 7–8,>8 h), vigorous activity (yes, no), breastfeeding length (months, continuous), dietary behavior change (yes, no), and alcohol intake (yes, no)*.

*** *Model 3 additionally adjusted for BMI (<20, 20– <23, 23– <25, ≥ 25 kg/m^2^)*.

In the stratified analysis, the association between MDD-W scores and obesity was not modified by age nor length of residence in Korea (Table 5). Although, the association was not statistically significant, we found a more pronounced association for DDII and LDL-cholesterol among the participants under age 35 compared to the participants above age 35 (Table 6). We also found a suggestive pronounced association with LDL cholesterol among the participants with <8 years of residence in Korea compared to the immigrants who moved earlier. The associations of DDII with triglycerides and HOMA-IR did not differ by age, BMI, or length of stay in Korea (Supplementary Table 9).

**Table 5:** Subgroup analysis of the association of MDD-W with waist circumferences in the FiLWHEL study.

	MDD-W^*^			
	**≤4**	**5**	**≥6**	***p-*interaction**
**Age, years**				0.855
<35	1	0.79 (0.37–1.68)	0.60 (0.29–1.25)	
Case/total number	(35/90)	(16/47)	(18/64)	
≥35	1	1.00 (0.48–2.08)	0.59 (0.31–1.14)	
Case/total number	(47/86)	(27/49)	(34/75)	
**Body mass index, kg/m**^**2**^				**–**
<25	1	0.62 (0.28–1.36)	0.51 (0.26–1.01)	
Case/total number	(31/119)	(12/64)	(20/105)	
≥25^†^	1	-	-	
Case/total number	(51/57)	(31/32)	(32/34)	
**Length of residence, years**				0.593
≤ 8	1	0.73 (0.36–1.50)	0.68 (0.35–1.32)	
Case/total number	(40/104)	(17/52)	(25/74)	
>8	1	1.08 (0.49–2.40)	0.50 (0.25–1.01)	
Case/total number	(42/72)	(26/44)	(27/65)	

*MDD-W, Minimum Dietary Diversity for Women; FiLWHEL, Filipino Women's Diet and Health Study; BMI, Body mass index*.

* *Adjusted for age (years, continuous). energy intake (kcal, continuous), dietary behavior changes (yes, no), employment status (yes, no), smoking cohabitant (yes, no), region (urban, rural), and BMI (<20, 20– <23, 23– <25, ≥ 25 kg/m^2^)*.

† *Data not shown as the analysis was not performed due to the limited number of non-cases*.

**Table 6:** Subgroup analysis of the association of DDII with low-density lipoprotein cholesterols in the FiLWHEL study.

	DDII^**^		
	**Q1–3**	**Q4,5**	***p-*interaction**
**Age, years**			0.062
<35	1	6.55 (2.39–17.93)	
Case/total number	(11/159)	(14/43)	
≥35	1	1.60 (0.74–3.46)	
Case/total number	(16/88)	(35/115)	
**Body mass index, kg/m**^**2**^			0.467
<25	1	1.11 (0.43–2.88)	
Case/total number	(25/227)	(12/58)	
≥25	1	3.10 (0.53–18.12)	
Case/total number	(2/20)	(37/100)	
**Length of residence, years**			0.153
≤ 8	1	3.83 (1.66–8.83)	
Case/total number	(16/171)	(21/59)	
>8	1	1.96 (0.81–4.72)	
Case/total number	(11/76)	(28/99)	

*DDII, Data Derived Inflammatory Index; FiLWHEL, Filipino Women's Diet and Health Study; BMI, Body mass index*.

** *Adjusted for age (years, continuous). energy intake (kcal, continuous), education level (high school or less, associate/vocational, college or above), sleep hours (<5, 5–6, 7–8,>8 h), vigorous activity (yes, no), breastfeeding length (months, continuous), dietary behavior change (yes, no), and alcohol intake (yes, no)*.

In the sensitivity analysis for the quartiles of MDD-W using the logistic regression, the trend remained statistically significant for triglycerides, total cholesterol, and TG/HDL ratio (Supplementary Table 2). When MDD-W was examined using the GLM procedure, we found a suggestive inverse trend between lower means of waist circumferences with higher scores of MDD-W (*p* for trend = 0.052 for 3 categories and 0.071 for 4 categories) (Supplementary Tables 3, 4). The association was retained in the sensitivity analysis of dyslipidemia and insulin resistance by quintiles of DDII using both logistic regression and GLM procedure (Supplementary Tables 5, 6). When we limited the analysis to WRA, we found increased odds of triglycerides, total cholesterol, and TG/HDL ratio (Supplementary Table 7). A similar trend was found for abdominal obesity, total cholesterol, TG/HDL ratio, insulin levels, and HOMA-IR with hsCRP levels (Supplementary Table 8). We found a similar association for MDD-W and DDII, compared to the main results, when we adjusted for BMI as a continuous variable (Supplementary Tables 10, 11).

## Discussion

In this analysis of 413 Filipino immigrant women in Korea, we found a statistically significant association between greater dietary diversity and decreased prevalence of abdominal obesity and increased prevalence of dyslipidemia. We also found a statistically significant association between increased inflammatory index and increased prevalence of dyslipidemia and insulin resistance.

Higher points of MDD-W score were inversely associated with central obesity, which corresponded with previous epidemiological studies on dietary diversity and chronic disease (48–50). A cross-sectional study examining 289 Iranian female participants found a statistically significant inverse association between quartiles of DDS and obesity and abdominal adiposity; ORs (95% CIs): 1.00, 0.43 (0.14, 1.39), 0.34 (0.11, 1.29), and 0.25 (0.09, 0.98) for obesity (*p* for trend = 0.04), and ORs (95% CIs): 1.00, 0.64 (0.32, 1.65), 0.45 (0.21, 1.09), and 0.28 (0.11, 1.12) for abdominal adiposity (*p* for trend = 0.04) (51). A prior case-cohort study from European Prospective Investigation into Cancer and Nutrition-Norfolk study found lower risk of T2DM among participants consuming greater variety of fruit (RR: 0.70, 95% CI: 0.53, 0.91), vegetable (RR: 0.77, 95% CI: 0.61, 0.98), and fruits and vegetables combined (RR: 0.61, 95% CI: 0.48, 0.78) (52). Indeed, consuming a variety of food types may be beneficial for sustaining healthy dietary patterns and preventing non-communicable diseases (4, 53). Various international and national guidelines recommend diverse nutrient-dense food consumption across all food groups, with major emphasis on vegetables and protein sources (3, 54, 55).

We found that greater dietary diversity was associated with higher prevalence of hypercholesterolemia. Despite the accumulating literature supporting the association between dietary diversity and obesity, limited evidence exists regarding the lipid profiles (12, 56–58). In the cross-sectional analysis of the 2016 Chinese Urban Adults Diet and Health Study (CUADHS), adherence to Chinese healthy food diversity (HFD) recommendations was not significantly associated with metabolic syndrome and its components after adjusted for potential confounders (59). A U.S. study examining the risk of coronary heart disease using the data from three cohort studies did not observe a significant association with MDD-W (28). Although, the reason we observed high total cholesterol levels with MDD-W scores remains unclear, other dietary indices, including Healthy Eating Index (HEI), Alternate Healthy Eating Index (AHEI), MedDietScore (MDS), and Dietary Approaches to Stop Hypertension (DASH), may be more relevant for CVD-related lipid profiles (60). A report from the Multi-Ethnic Study of Atherosclerosis (MESA) reported a weak correlation between DDS and diet quality scores such as DASH and the AHEI (58). We also observed that MDD-W scores were not correlated with DDII scores in our study population.

Recently, diet quality scores reflecting the diet-related inflammation status have been developed internationally, which showed a decent agreement with previously established measurement schemes (20, 61–63). We found a statistically significant association between proinflammatory DDII scores and increased prevalence of dyslipidemia and insulin resistance in our study. Our findings aligned with recent epidemiological literature investigating the association between dietary inflammation estimates and chronic disease (64–66). A study examining the literature-derived dietary inflammatory index (DII®), consisting of 46 pro- and anti-inflammatory parameters, found a statistically significant association between higher DII® and metabolic syndrome (65).

The inflammatory status induced by surplus energy intake may play a role in the pathophysiology linking diet, obesity, and dyslipidemia (67). The chronic inflammation state of obesity is widely acknowledged as a pivotal contributor to the progression of dyslipidemia, T2DM, CVD, and certain cancers (68, 69). Excess energy mainly stored in adipocytes triggers obesity-associated inflammation through multiple pathways, including inflammatory cytokine upregulation, increased oxidative stress, and low-grade inflammatory response (70, 71). Subsequently, the inflammation status yields the development of insulin resistance and increased IGF-1 (72). Moreover, hypercaloric intake mainly from carbohydrate sources is linked to greater stores of intrahepatic triglycerides, resulting in atherogenic dyslipidemia (73).

Immigrants alter their dietary behaviors as part of their acculturation process, known as “dietary acculturation” (74, 75). It involves a complex multidimensional interaction between cultural, social, economic, and environmental factors, with variations among individuals. In our previous cross-sectional analysis, we found that Filipino immigrants consume a smaller variety of foods compared with Koreans (76). A study examining the dietary diversity of Filipino children from the 2013 Filipino National Nutrition Survey found that mean (± SD) DDS score was 4.1 (± 1.3) out of nine food groups (77). Moreover, another Filipino pediatric study reported that the meals are mainly composed of unhealthy sources (refined rice or cookies), while vegetables, fruits, and meats contribute less to daily nutrient intake (78). With suggestions that maintaining the diet pattern low in food diversity may not be optimal for Filipino immigrants, favorable acculturation with food diversity may be an option. Further, epidemiological investigations are warranted regarding dietary acculturation and health outcomes among Filipino immigrants in Korea.

The investigation of Filipino immigrant women in Korea is unique to the literature; to the best of our knowledge, this is the first study to examine the association between diet quality scores and prevalence of obesity, dyslipidemia, and insulin resistance in the immigrant population of East Asia. The strengths of the current study are that it focuses on immigrant Filipino women in the multicultural society of South Korea and that the data collection methodology used across sites was standardized and uniform, along with centralized sample processing. However, several limitations need to be acknowledged. First, the cross-sectional nature of the study limits our ability to examine temporal or causal relationships. In addition, dietary data were collected through a single-day 24-h recall, which may not reflect the usual daily intake. The study was limited in power due to its relatively small sample size. Moreover, although we controlled for potential confounding factors, the results are still subject to residual confounding.

In conclusion, greater dietary diversity was positively associated with a lower prevalence of obesity, but higher prevalence of dyslipidemia, while proinflammatory patterns were associated with an elevated prevalence of dyslipidemia and insulin resistance. Further epidemiological and clinical studies are warranted to replicate our findings.

## Data Availability Statement

The raw data supporting the conclusions of this article will be made available by the authors, without undue reservation.

## Ethics Statement

The studies involving human participants were reviewed and approved by Institutional Review Board of Sookmyung Women's University. The patients/participants provided their written informed consent to participate in this study.

## Author Contributions

HK outlined the initial manuscript and performed data analysis. HL and MK assisted in the manuscript drafting. CL and JL conceptualized, supervised the study process, and received funding for the project. HK, HL, SP, GC, SH, SY, SL, and JL were involved in the data collection and critically revised the intellectual content of the manuscript. All authors made a significant contribution to the interpretation of the data.

## Conflict of Interest

The authors declare that the research was conducted in the absence of any commercial or financial relationships that could be construed as a potential conflict of interest.
