# Supplementary material for: Association Between Diet Quality and Prevalence of Obesity, Dyslipidemia, and Insulin Resistance Among Filipino Immigrant Women in Korea: The Filipino Women's Diet and Health Study
Source: Front Public Health. 2021 Jul 1;9:647661. doi: 10.3389/fpubh.2021.647661 (PMC8281297; doi:10.3389/fpubh.2021.647661)
Supplement: Supplementary file 1 [file Data_Sheet_1.pdf]

Supplementary Table 1. Least squares means (95% confidence interval) of high sensitivity C- reactive protein according to the quintiles of DDII in the FiLWHEL study.

	LS-Means (95%CI)					
**(n=371)**	**Q1**	**Q2**	**Q3**	**Q4**	**Q5**	***p-trend***
**High sensitivity C- reactive protein**						
**Age and energy adjusted**	0.79 (0.61-1.02)	0.96 (0.76-1.22)	1.02 (0.82-1.28)	1.14 (0.90-1.45)	1.65 (1.30-2.09)	<.001

Supplementary Table 2. Odds ratios (ORs) and 95% confidence intervals (CIs) of BMI, Waist Circumferences, Triglycerides, HDL-C, LDL-C, Total Cholesterols, TG/HDL, HbA1c, Fasting Blood Glucose, Insulin, HOMA-IR, according to the quartiles of MDD-W scoring in the FiLWHEL study.

	**ORs (95%CIs)** according to the quartiles of MDD-W				
	**≤3**	**4**	**5**	**≥6**	***p-trend***
Body Mass Index (≥25kg/m^2)					
Case/Total number	(23/83)	(34/96)	(32/95)	(35/139)	
Model 1*	1	1.51 (0.79 - 2.88)	1.33 (0.70 - 2.55)	0.84 (0.45 - 1.57)	0.334
Model 2**	1	1.51 (0.79 - 2.89)	1.31 (0.68 - 2.53)	0.81 (0.44 - 1.52)	0.278
Waist Circumferences (≥80cm)					
Case/Total number	(35/82)	(47/94)	(43/96)	(52/139)	
Model 1*	1	1.38 (0.75 - 2.56)	1.05 (0.57 - 1.94)	0.71 (0.40 - 1.27)	0.096
Model 2**	1	1.39 (0.75 - 2.60)	1.06 (0.57 - 1.98)	0.69 (0.39 - 1.24)	0.083
Model 3†	1	1.20 (0.47 - 3.07)	0.76 (0.31 - 1.84)	0.61 (0.27 - 1.40)	0.133
Triglycerides (≥150 mg/dL)					
Case/Total number	(3/83)	(10/94)	(7/96)	(19/132)	
Model 1*	1	3.29 (0.86 - 12.54)	2.08 (0.52 - 8.36)	4.38 (1.25 - 15.37)	0.032
Model 2††	1	3.41 (0.87 - 13.40)	1.91 (0.46 - 7.96)	5.30 (1.46 - 19.24)	0.016
High Density Lipoprotein Cholesterol (<40 mg/dL)					
Case/Total number	(3/83)	(8/94)	(3/96)	(14/132)	
Model 1*	1	2.38 (0.61 - 9.32)	0.85 (0.17 - 4.32)	3.21 (0.89 - 11.56)	0.117
Model 2††	1	2.28 (0.56 - 9.26)	0.75 (0.14 - 3.93)	3.68 (0.99 - 13.70)	0.076
Low Density Lipoprotein Cholesterol (≥130 mg/dL)					
Case/Total number	(17/83)	(15/94)	(16/96)	(28/132)	
Model 1*	1	0.76 (0.34 - 1.66)	0.76 (0.35 - 1.64)	1.00 (0.50 - 1.98)	0.880
Model 2††	1	0.72 (0.31 - 1.65)	0.74 (0.33 - 1.70)	1.18 (0.57 - 2.44)	0.522
Total Cholesterol (≥200 mg/dL)					

Case/Total number	(20/83)	(20/94)	(23/96)	(46/132)	
Model 1[*]	1	0.88 (0.43 - 1.82)	0.98 (0.48 - 1.98)	1.64 (0.87 - 3.08)	0.068
Model 2[††]	1	0.87 (0.41 - 1.87)	0.97 (0.46 - 2.04)	1.88 (0.97 - 3.64)	0.030
TG/HDL (≥3)					
Case/Total number	(3/83)	(10/94)	(8/96)	(22/132)	
Model 1[*]	1	3.05 (0.81 - 11.54)	2.35 (0.60 - 9.20)	5.23 (1.51 - 18.12)	0.007
Model 2[††]	1	3.23 (0.82 - 12.74)	2.44 (0.60 - 9.95)	7.12 (1.96 - 25.88)	0.002
HbA1c (≥5.7%)					
Case/Total number	(27/83)	(31/94)	(37/96)	(46/132)	
Model 1[*]	1	1.16 (0.59 - 2.24)	1.37 (0.71 - 2.63)	1.07 (0.58 - 1.98)	0.819
Model 2[††]	1	1.13 (0.57 - 2.24)	1.26 (0.64 - 2.46)	1.12 (0.60 - 2.10)	0.715
Fasting blood glucose (≥100 mg/dL)					
Case/Total number	(9/83)	(10/94)	(11/96)	(13/132)	
Model 1[*]	1	1.13 (0.42 - 3.05)	1.08 (0.41 - 2.88)	0.86 (0.34 - 2.18)	0.687
Model 2[††]	1	1.01 (0.36 - 2.82)	1.01 (0.37 - 2.77)	0.84 (0.32 - 2.17)	0.694
Insulin (≥ 15 mg/dL)					
Case/Total number	(9/83)	(14/94)	(13/96)	(21/132)	
Model 1[*]	1	1.45 (0.59 - 3.59)	1.34 (0.54 - 3.35)	1.70 (0.73 - 3.95)	0.267
Model 2[††]	1	1.38 (0.53 - 3.65)	1.38 (0.52 - 3.65)	2.04 (0.82 - 5.07)	0.127
HOMA-IR (≥3.16)					
Case/Total number	(12/83)	(15/94)	(15/96)	(23/132)	
Model 1[*]	1	1.15 (0.50 - 2.64)	1.12 (0.49 - 2.56)	1.28 (0.60 - 2.74)	0.551
Model 2[††]	1	1.07 (0.44 - 2.60)	1.09 (0.45 - 2.62)	1.47 (0.65 - 3.33)	0.332

Abbreviations : FiLWHEL, Filipino Women's Diet and Health Study; MDD-W, Minimum Dietary Diversity for Women; HDL-C, High Density Lipoprotein Cholesterol; LDL-C, Low Density Lipoprotein Cholesterol; HbA1c, Hemoglobin A1C; BMI, Body mass index; TG/HDL, Triglycerides/High density lipoprotein cholesterols; HOMA-IR, Homeostasis Model Assessment of Insulin Resistance.

[*]Model 1 adjusted for age(years, continuous) and energy intake(kcal, continuous).

[**]Model 2 additionally adjusted for dietary behavior changes (yes, no), employment status(yes, no), smoking cohabitant (yes, no), and region (urban, rural).

†Model 3 additionally adjusted for dietary behavior changes (yes, no), employment status(yes, no), smoking cohabitant (yes, no), region (urban, rural), and BMI (<20, 20-<23, 23-<25, ≥25 kg/m^2).
††Model 2 additionally adjusted for dietary behavior changes (yes, no), employment status(yes, no), smoking cohabitant (yes, no), region (urban, rural), and BMI (<20, 20-<23, 23-<25, ≥25 kg/m^2).

Supplementary Table 3. Least squares means (LS-Means) and 95% confidence intervals (CIs) of BMI, Waist Circumferences, Triglycerides, HDL-C, LDL-C, Total Cholesterols, TG/HDL, HbA1c, Fasting Blood Glucose, Insulin, HOMA-IR, according to the tertiles of MDD-W scoring in the FiLWHEL study.

	**LS-Means (95%CI)** according to the tertiles of MDD-W			
	**≤4**	**5**	**≥6**	***p-trend***
	**179**	**95**	**139**	
Body Mass Index (kg/m^2)†				
Model 1*	23.44 (22.91 - 23.97)	23.87 (23.14 - 24.63)	22.92 (22.33 - 23.52)	0.242
Model 2**	23.40 (22.87 - 23.95)	23.80 (23.06 - 24.56)	22.85 (22.25 - 23.46)	0.202
Waist Circumferences (cm)†				
Model 1*	79.45 (78.14 - 80.77)	80.09 (78.32 - 81.90)	77.52 (76.09 - 78.98)	0.067
Model 2**	79.27 (77.97 - 80.60)	79.89 (78.10 - 81.71)	77.25 (75.80 - 78.72)	0.052
Model 3†	77.93 (77.10 - 78.76)	78.02 (76.89 - 79.16)	77.03 (76.09 - 77.97)	0.167
Triglycerides (mg/dL)				
Model 1*	77.90 (72.78 - 83.37)	78.09 (71.22 - 85.63)	81.03 (74.90 - 87.67)	0.469
Model 2††	75.13 (70.37 - 80.21)	74.44 (68.09 - 81.39)	80.12 (74.21 - 86.49)	0.223
High Density Lipoprotein Cholesterol (mg/dL)				
Model 1*	56.72 (54.76 - 58.74)	57.50 (54.83 - 60.31)	55.19 (53.00 - 57.48)	0.355
Model 2††	57.48 (55.54 - 59.48)	58.51 (55.84 - 61.30)	55.02 (52.86 - 57.27)	0.123
Low Density Lipoprotein Cholesterol (130 mg/dL)				
Model 1*	97.36 (92.96 - 101.97)	97.91 (91.95 - 104.25)	102.13 (96.80 - 107.75)	0.197
Model 2††	95.36 (91.12 - 99.79)	95.79 (90.03 - 101.91)	101.41 (96.16 - 106.95)	0.088
Total Cholesterol (mg/dL)				
Model 1*	174.61 (169.74 - 179.62)	175.18 (168.59 - 182.03)	178.78 (173.02 - 184.73)	0.296
Model 2††	172.57	173.03	177.54	0.203

	(167.78 - 177.50)	(166.51 - 179.80)	(171.79 - 183.49)	
TG/HDL				
Model 1*	1.37 (1.26 - 1.50)	1.36 (1.20 - 1.53)	1.47 (1.32 - 1.63)	0.359
Model 2††	1.31 (1.20 - 1.42)	1.27 (1.13 - 1.43)	1.46 (1.32 - 1.61)	0.117
HbA1c (%)				
Model 1*	5.47 (5.40 - 5.55)	5.51 (5.41 - 5.62)	5.43 (5.34 - 5.52)	0.537
Model 2††	5.45 (5.37 - 5.53)	5.47 (5.37 - 5.58)	5.42 (5.33 - 5.51)	0.684
Fasting blood glucose (mg/dL)				
Model 1*	88.20 (86.72 - 89.71)	87.91 (85.91 - 89.96)	87.42 (85.71 - 89.15)	0.500
Model 2††	87.65 (86.18 - 89.15)	87.47 (85.47 - 89.52)	87.01 (85.30 - 88.76)	0.581
Insulin (mg/dL)				
Model 1*	7.85 (7.15 - 8.62)	7.8 (6.87 - 8.85)	7.94 (7.13 - 8.85)	0.887
Model 2††	7.39 (6.80 - 8.03)	7.16 (6.40 - 8.02)	7.82 (7.10 - 8.62)	0.417
HOMA-IR				
Model 1*	1.71 (1.54 - 1.89)	1.69 (1.47 - 1.94)	1.71 (1.52 - 1.93)	0.985
Model 2††	1.60 (1.46 - 1.75)	1.55 (1.37 - 1.75)	1.68 (1.51 - 1.87)	0.524

Abbreviations : FiLWHEL, Filipino Women's Diet and Health Study; MDD-W, Minimum Dietary Diversity for Women; HDL-C, High Density Lipoprotein Cholesterol; LDL-C, Low Density Lipoprotein Cholesterol; HbA1c, Hemoglobin A1C; BMI, Body mass index; TG/HDL, Triglycerides/High density lipoprotein cholesterols; HOMA-IR, Homeostasis Model Assessment of Insulin Resistance.

*Model 1 adjusted for age(years, continuous) and energy intake(kcal, continuous).

**Model 2 additionally adjusted for dietary behavior changes (yes, no), employment status(yes, no), smoking cohabitant (yes, no), and region (urban, rural).

†Model 3 additionally adjusted for dietary behavior changes (yes, no), employment status(yes, no), smoking cohabitant (yes, no), region (urban, rural), and BMI (<20, 20-<23, 23-<25, ≥25 kg/m^2).

††Model 2 additionally adjusted for dietary behavior changes (yes, no), employment status(yes, no), smoking cohabitant (yes, no), region (urban, rural), and BMI (<20, 20-<23, 23-<25, ≥25 kg/m^2).

Supplementary Table 4. Least squares means (LS-Means) and 95% confidence intervals (CIs) of BMI, Waist Circumferences, Triglycerides, HDL-C, LDL-C, Total Cholesterols, TG/HDL, HbA1c, Fasting Blood Glucose, Insulin, HOMA-IR, according to the quartiles of MDD-W scoring in the FiLWHEL study.

	**LS-Means (95%CI) according to the quartiles of MDD-W**				
	**≤3**	**4**	**5**	**≥6**	***p-trend***
	**83**	**96**	**95**	**139**	
Body Mass Index (kg/m²)†					
Model 1*	23.43 (22.66 - 24.23)	23.44 (22.72 - 24.18)	23.87 (23.14 - 24.63)	22.92 (22.33 - 23.52)	0.332
Model 2**	23.40 (22.63 - 24.20)	23.41 (22.69 - 24.15)	23.80 (23.06 - 24.56)	22.85 (22.25 - 23.46)	0.281
Waist Circumferences (cm)†					
Model 1*	79.70 (77.79 - 81.66)	79.23 (77.45 - 81.05)	80.09 (78.32 - 81.90)	77.52 (76.09 - 78.98)	0.087
Model 2**	79.50 (77.59 - 81.46)	79.07 (77.30 - 80.88)	79.89 (78.10 - 81.71)	77.25 (75.80 - 78.72)	0.071
Model 3†	78.24 (77.02 - 79.48)	77.66 (76.54 - 78.79)	78.02 (76.90 - 79.16)	77.04 (76.10 - 77.98)	0.149
Triglycerides (mg/dL)					
Model 1*	79.00 (71.53 - 87.25)	76.93 (70.07 - 84.48)	78.09 (71.21 - 85.63)	81.04 (74.90 - 87.68)	0.582
Model 2†	76.20 (69.21 - 83.90)	74.22 (67.91 - 81.12)	74.46 (68.10 - 81.42)	80.16 (74.24 - 86.55)	0.328
High Density Lipoprotein Cholesterol (mg/dL)					
Model 1*	56.30 (53.48 - 59.26)	57.09 (54.40 - 59.92)	57.50 (54.83 - 60.31)	55.19 (52.99 - 57.49)	0.496
Model 2††	56.89 (54.10 - 59.84)	57.98 (55.34 - 60.74)	58.49 (55.82 - 61.29)	55.00 (52.84 - 57.26)	0.236
Low Density Lipoprotein Cholesterol (130 mg/dL)					
Model 1*	98.09 (91.67 - 104.96)	96.72 (90.75 - 103.08)	97.91 (91.95 - 104.26)	102.13 (96.79 - 107.76)	0.272
Model 2††	95.89 (89.69 - 102.52)	94.91 (89.23 - 100.96)	95.80 (90.04 - 101.93)	101.43 (96.17 - 106.98)	0.134
Total Cholesterol (mg/dL)					

Model 1*	175.12 (168.03 - 182.52)	174.16 (167.51 - 181.07)	175.18 (168.58 - 182.03)	178.78 (173.01 - 184.74)	0.365
Model 2††	172.69 (165.69 - 180)	172.47 (166.00 - 179.20)	173.03 (166.51 - 179.81)	177.55 (171.78 - 183.50)	0.248
TG/HDL					
Model 1*	1.40 (1.23 - 1.60)	1.35 (1.19 - 1.52)	1.36 (1.20 - 1.53)	1.47 (1.32 - 1.63)	0.491
Model 2††	1.34 (1.18 - 1.52)	1.28 (1.14 - 1.44)	1.27 (1.13 - 1.43)	1.46 (1.32 - 1.61)	0.216
HbA1c (%)					
Model 1*	5.48 (5.36 - 5.60)	5.47 (5.36 - 5.58)	5.51 (5.41 - 5.62)	5.43 (5.34 - 5.52)	0.592
Model 2††	5.46 (5.35 - 5.58)	5.44 (5.33 - 5.55)	5.47 (5.37 - 5.58)	5.42 (5.33 - 5.52)	0.674
Fasting blood glucose (mg/dL)					
Model 1*	88.03 (85.87 - 90.24)	88.36 (86.32 - 90.44)	87.91 (85.91 - 89.96)	87.42 (85.71 - 89.15)	0.567
Model 2††	87.41 (85.25 - 89.62)	87.86 (85.86 - 89.91)	87.47 (85.47 - 89.52)	87.00 (85.29 - 88.75)	0.668
Insulin (mg/dL)					
Model 1*	8.01 (6.99 - 9.19)	7.71 (6.78 - 8.77)	7.80 (6.87 - 8.85)	7.94 (7.13 - 8.85)	0.997
Model 2††	7.63 (6.75 - 8.62)	7.20 (6.43 - 8.06)	7.17 (6.40 - 8.03)	7.83 (7.10 - 8.63)	0.622
HOMA-IR					
Model 1*	1.74 (1.50 - 2.02)	1.68 (1.46 - 1.93)	1.69 (1.47 - 1.94)	1.71 (1.52 - 1.93)	0.927
Model 2††	1.65 (1.44 - 1.88)	1.56 (1.38 - 1.77)	1.55 (1.37 - 1.75)	1.68 (1.51 - 1.87)	0.712

Abbreviations : FiLWHEL, Filipino Women's Diet and Health Study; MDD-W, Minimum Dietary Diversity for Women; HDL-C, High Density Lipoprotein Cholesterol; LDL-C, Low Density Lipoprotein Cholesterol; HbA1c, Hemoglobin A1C; BMI, Body mass index; TG/HDL, Triglycerides/High density lipoprotein cholesterols; HOMA-IR, Homeostasis Model Assessment of Insulin Resistance.

*Model 1 adjusted for age(years, continuous) and energy intake(kcal, continuous).

**Model 2 additionally adjusted for dietary behavior changes (yes, no), employment status(yes, no), smoking cohabitant (yes, no), and region (urban, rural).

†Model 3 additionally adjusted for dietary behavior changes (yes, no), employment status(yes, no), smoking cohabitant (yes, no), region (urban, rural), and BMI (<20, 20-<23, 23-<25, ≥25 kg/m^2).

††Model 2 additionally adjusted for dietary behavior changes (yes, no), employment status(yes, no), smoking cohabitant (yes, no), region (urban, rural), and BMI (<20, 20-<23, 23-<25, ≥25 kg/m^2).

Supplementary Table 5. Odds ratios (ORs) and 95% confidence intervals (CIs) of Triglycerides, HDL-C, LDL-C, Total Cholesterols, TG/HDL, HbA1c, Fasting Blood Glucose, Insulin, HOMA-IR, and Waist Circumferences according to DDII in the FiLWHEL study.

	**ORs (95%CIs) according to quintiles of DDII**					
	**Q1**	**Q2**	**Q3**	**Q4**	**Q5**	***p-trend***
**(n=405)**	82	83	82	77	81	
Triglycerides (≥150 mg/dL)						
Case/Total number	(3/82)	(4/83)	(5/82)	(8/77)	(19/81)	
Model 1*	1	1.32 (0.28 - 6.13)	1.69 (0.38 - 7.43)	3.00 (0.73 - 12.39)	7.88 (2.02 - 30.72)	<.001
High Density Lipoprotein Cholesterol (<40 mg/dL)						
Case/Total number	(3/82)	(5/83)	(6/82)	(6/77)	(8/81)	
Model 1*	1	1.87 (0.43 - 8.17)	2.46 (0.58 - 10.45)	2.99 (0.68 - 13.22)	4.33 (0.98 - 19.08)	0.040
Low Density Lipoprotein Cholesterol (≥130 mg/dL)						
Case/Total number	(6/82)	(13/83)	(8/82)	(18/77)	(31/81)	
Model 1*	1	2.19 (0.78 - 6.12)	1.22 (0.40 - 3.75)	3.19 (1.14 - 8.94)	6.12 (2.20 - 16.98)	<.001
Total Cholesterol (≥200 mg/dL)						
Case/Total number	(9/82)	(19/83)	(15/82)	(26/77)	(40/81)	
Model 1*	1	2.20 (0.92 - 5.25)	1.57 (0.64 - 3.90)	3.25 (1.34 - 7.83)	5.79 (2.39 - 14.04)	<.001
TG/HDL (≥3)						
Case/Total number	(3/82)	(3/83)	(9/82)	(10/77)	(18/81)	
Model 1*	1	1.05 (0.20 - 5.39)	3.63 (0.93 - 14.22)	4.84 (1.22 - 19.28)	10.10 (2.59 - 39.45)	<.001
HbA1c (≥5.7%)						
Case/Total number	(18/82)	(18/83)	(25/82)	(31/77)	(49/81)	
Model 1*	1	0.85 (0.40 - 1.82)	1.26 (0.61 - 2.62)	1.60 (0.76 - 3.41)	3.33 (1.55 - 7.14)	<.001

Fasting blood glucose (≥100 mg/dL)						
Case/Total number	(1/82)	(5/83)	(9/82)	(9/77)	(19/81)	
Model 1*	1	4.35 (0.49 - 38.46)	7.59 (0.92 - 62.44)	6.37 (0.75 - 53.80)	12.98 (1.59 - 106.08)	0.003
Insulin (≥ 15 mg/dL)						
Case/Total number	(3/82)	(7/83)	(11/82)	(16/77)	(20/81)	
Model 1*	1	3.48 (0.85 - 14.24)	7.07 (1.84 - 27.17)	17.91 (4.66 - 68.84)	31.15 (7.89 – 123.00)	<.001
HOMA-IR (≥3.16)						
Case/Total number	(3/82)	(8/83)	(12/82)	(15/77)	(27/81)	
Model 1*	1	3.57 (0.90 - 14.14)	6.44 (1.71 - 24.22)	11.49 (3.04 - 43.42)	30.01 (7.90 - 113.97)	<.001

Abbreviations : FiLWHEL, Filipino Women's Diet and Health Study; DDII, Data Derived Inflammatory Index; HDL-C, High Density Lipoprotein Cholesterol; LDL-C, Low Density Lipoprotein Cholesterol; HbA1c, Hemoglobin A1c; BMI, Body mass index; TG/HDL, Triglycerides/High density lipoprotein cholesterols; HOMA-IR, Homeostasis Model Assessment of Insulin Resistance.

*Model 1 adjusted for age(years, continuous) and energy intake(kcal, continuous).

Supplementary Table 6. Least squares means (LS-Means) and 95% confidence intervals (CIs) of Triglycerides, HDL-C, LDL-C, Total Cholesterols, TG/HDL, HbA1c, Fasting Blood Glucose, Insulin, HOMA-IR, and Waist Circumferences according to DDII in the FiLWHEL study.

	**LS-Means (95%CI) according to quintiles of DDII**					
**(n=405)**	**Q1**	**Q2**	**Q3**	**Q4**	**Q5**	***p-trend***
Triglycerides (mg/dL)						
Model 1*	63.87 (57.72 - 70.68)	72.90 (66.24 - 80.24)	77.44 (70.39 - 85.20)	84.15 (76.13 - 93.01)	101.91 (92.02 - 112.86)	<.001
Model 2**	63.47 (56.44 - 71.38)	71.51 (63.54 - 80.48)	76.56 (68.61 - 85.42)	83.32 (73.88 - 93.97)	101.22 (90.05 - 113.77)	<.001
High Density Lipoprotein Cholesterol (mg/dL)						
Model 1*	60.17 (57.06 - 63.45)	60.79 (57.81 - 63.92)	53.95 (51.32 - 56.72)	56.44 (53.56 - 59.48)	51.13 (48.47 - 53.95)	<.001
Model 2**	60.18 (56.58 - 64.01)	59.97 (56.36 - 63.81)	53.60 (50.60 - 56.78)	55.41 (52.01 - 59.02)	50.47 (47.46 - 53.66)	<.001
Low Density Lipoprotein Cholesterol (mg/dL)						
Model 1*	89.49 (83.34 - 96.09)	95.42 (89.21 - 102.07)	99.48 (93.03 - 106.38)	103.35 (96.33 - 110.88)	108.90 (101.36 - 116.99)	<.001
Model 2**	88.41 (81.49 - 95.92)	93.13 (85.79 - 101.09)	98.73 (91.50 - 106.53)	101.96 (93.79 - 110.84)	107.19 (98.84 - 116.25)	<.001
Total Cholesterol (mg/dL)						
Model 1*	165.87 (158.81 - 173.25)	175.25 (168.18 - 182.61)	173.37 (166.41 - 180.63)	181.46 (173.82 - 189.43)	185.62 (177.66 - 193.94)	0.001

Model 2**	165.30 (157.21 - 173.80)	172.01 (163.55 - 180.92)	172.36 (164.48 - 180.62)	178.92 (169.96 - 188.35)	183.03 (174.12 - 192.40)	0.002
TG/HDL (≥3)						
Model 1*	1.06 (0.93 - 1.21)	1.20 (1.06 - 1.36)	1.44 (1.27 - 1.63)	1.49 (1.31 - 1.70)	1.99 (1.74 - 2.28)	<.001
Model 2**	1.05 (0.91 - 1.23)	1.19 (1.02 - 1.39)	1.43 (1.24 - 1.65)	1.50 (1.29 - 1.76)	2.01 (1.72 - 2.33)	<.001
HbA1c (%)						
Model 1*	5.39 (5.28 - 5.51)	5.33 (5.22 - 5.44)	5.41 (5.30 - 5.53)	5.45 (5.33 - 5.57)	5.78 (5.66 - 5.91)	<.001
Model 2**	5.37 (5.23 - 5.51)	5.33 (5.19 - 5.47)	5.40 (5.28 - 5.53)	5.46 (5.32 - 5.60)	5.78 (5.63 - 5.92)	<.001
(Case=141)						
Fasting blood glucose (mg/dL)						
Model 1*	85.37 (83.16 - 87.63)	87.42 (85.29 - 89.61)	87.80 (85.66 - 89.99)	87.82 (85.58 - 90.12)	91.11 (88.75 - 93.55)	0.002
Model 2**	84.44 (81.95 - 87.02)	85.90 (83.35 - 88.53)	86.86 (84.46 - 89.32)	86.43 (83.81 - 89.12)	89.89 (87.25 - 92.62)	0.003
Insulin (mg/dL)						
Model 1*	4.66 (4.10 - 5.28)	6.27 (5.56 - 7.06)	8.36 (7.43 - 9.42)	9.44 (8.33 - 10.69)	13.37 (11.78 - 15.19)	<.001
Model 2**	4.57 (3.94 - 5.29)	6.09 (5.25 - 7.07)	8.20 (7.15 - 9.41)	9.25 (7.95 - 10.76)	13.11 (11.32 - 15.18)	<.001
HOMA-IR						
Model 1*	0.98 (0.85 - 1.13)	1.35 (1.19 - 1.54)	1.81 (1.59 - 2.06)	2.04 (1.78 - 2.34)	3.01 (2.61 - 3.46)	<.001
Model 2**	0.95 (0.81 - 1.12)	1.29 (1.1 - 1.52)	1.76 (1.51 - 2.04)	1.97 (1.67 - 2.33)	2.91 (2.48 - 3.41)	<.001

Abbreviations : FiLWHEL, Filipino Women's Diet and Health Study; DDII, Data Derived Inflammatory Index; HDL-C, High Density Lipoprotein Cholesterol; LDL-C, Low Density Lipoprotein Cholesterol; HbA1c, Hemoglobin A1c; BMI, Body mass index; TG/HDL, Triglycerides/High density lipoprotein cholesterols;

HOMA-IR, Homeostasis Model Assessment of Insulin Resistance.

*Model 1 adjusted for age(years, continuous) and energy intake(kcal, continuous).

**Model 2 additionally adjusted for education level (high school or less, associate/vocational, college or above), sleep hours (<5,5-6,7-8,>8 hours), vigorous activity (yes, no), breastfeeding length (months, continuous), dietary behavior change (yes, no), and alcohol intake (yes, no).

Supplementary Table 7. Odds ratios (ORs) and 95% confidence intervals (CIs) of BMI, Waist Circumferences, Triglycerides, HDL-C, LDL-C, Total Cholesterols, TG/HDL, HbA1c, Fasting Blood Glucose, Insulin, HOMA-IR, according to the MDD-W scoring for the WRA in FiLWHEL study.

	**ORs (95%CIs) according to tertiles of MDD-W**			
	**≤4**	**5**	**≥6**	***p-trend***
Body Mass Index (≥25kg/m^2)				
Case/Total number	(52/172)	(29/88)	(34/129)	
Model 1*	1	1.13 (0.65 - 1.97)	0.78 (0.47 - 1.31)	0.385
Model 2**	1	1.12 (0.64 - 1.97)	0.76 (0.45 - 1.28)	0.330
Waist Circumferences (≥80cm)				
Case/Total number	(76/169)	(39/89)	(48/129)	
Model 1*	1	0.94 (0.55 - 1.60)	0.65 (0.40 - 1.05)	0.087
Model 2**	1	0.96 (0.56 - 1.64)	0.63 (0.39 - 1.03)	0.074
Model 3†	1	0.74 (0.34 - 1.60)	0.59 (0.29 - 1.21)	0.145
Triglycerides (≥150 mg/dL)				
Case/Total number	(11/170)	(4/89)	(18/124)	
Model 1*	1	0.68 (0.21 - 2.21)	2.40 (1.09 - 5.30)	0.028
Model 2†	1	0.66 (0.20 - 2.19)	2.76 (1.20 - 6.34)	0.017
High Density Lipoprotein Cholesterol (<40 mg/dL)				
Case/Total number	(10/170)	(3/89)	(14/124)	
Model 1*	1	0.56 (0.15 - 2.09)	2.09 (0.89 - 4.90)	0.092
Model 2†	1	0.51 (0.13 - 1.94)	2.39 (0.98 - 5.81)	0.058
Low Density Lipoprotein Cholesterol (≥130 mg/dL)				
Case/Total number	(29/170)	(11/89)	(25/124)	
Model 1*	1	0.69 (0.33 - 1.46)	1.19 (0.65 - 2.16)	0.627
Model 2†	1	0.71 (0.32 - 1.57)	1.35 (0.72 - 2.55)	0.402
Total Cholesterol (≥200 mg/dL)				
Case/Total number	(35/170)	(18/89)	(41/124)	
Model 1*	1	0.98 (0.52 - 1.87)	1.85 (1.08 - 3.14)	0.027
Model 2†	1	0.98 (0.50 - 1.91)	2.05 (1.17 - 3.59)	0.014
TG/HDL (≥3)				
Case/Total number	(11/170)	(6/89)	(20/124)	
Model 1*	1	1.05 (0.37 - 2.93)	2.87 (1.31 - 6.26)	0.008
Model 2†	1	1.11 (0.38 - 3.25)	3.75 (1.61 - 8.74)	0.002
HbA1c (≥5.7%)				
Case/Total number	(53/170)	(32/89)	(41/124)	
Model 1*	1	1.27 (0.72 - 2.23)	1.00 (0.60 - 1.67)	0.947
Model 2†	1	1.16 (0.65 - 2.08)	1.05 (0.62 - 1.79)	0.830
Fasting blood glucose (≥100 mg/dL)				
Case/Total number	(16/170)	(10/89)	(11/124)	
Model 1*	1	1.28 (0.54 - 3.03)	0.85 (0.37 - 1.95)	0.746

Model 2†	1	1.27 (0.52 - 3.10)	0.78 (0.33 - 1.84)	0.617
Insulin (≥ 15 mg/dL)				
Case/Total number	(23/170)	(13/89)	(20/124)	
Model 1*	1	1.10 (0.53 - 2.31)	1.31 (0.68 - 2.53)	0.424
Model 2†	1	1.17 (0.53 - 2.57)	1.57 (0.77 - 3.23)	0.223
HOMA-IR (≥3.16)				
Case/Total number	(25/170)	(13/89)	(22/124)	
Model 1*	1	1.00 (0.48 - 2.06)	1.29 (0.69 - 2.43)	0.443
Model 2†	1	1.02 (0.47 - 2.22)	1.50 (0.75 - 2.97)	0.266

Abbreviations : FiLWHEL, Filipino Women's Diet and Health Study; MDD-W, Minimum Dietary Diversity for Women; HDL-C, High Density Lipoprotein Cholesterol; LDL-C, Low Density Lipoprotein Cholesterol; HbA1c, Hemoglobin A1C; BMI, Body mass index; TG/HDL, Triglycerides/High density lipoprotein cholesterols; HOMA-IR, Homeostasis Model Assessment of Insulin Resistance.

*Model 1 adjusted for age(years, continuous) and energy intake(kcal, continuous).

**Model 2 additionally adjusted for dietary behavior changes (yes, no), employment status(yes, no), smoking cohabitant (yes, no), and region (urban, rural).

†Model 3 additionally adjusted for dietary behavior changes (yes, no), employment status(yes, no), smoking cohabitant (yes, no), region (urban, rural), and BMI (<20, 20-<23, 23-<25, ≥25 kg/m^2).

††Model 2 additionally adjusted for dietary behavior changes (yes, no), employment status(yes, no), smoking cohabitant (yes, no), region (urban, rural), and BMI (<20, 20-<23, 23-<25, ≥25 kg/m^2).

Supplementary Table 8. Odds ratios (ORs) and 95% confidence intervals (CIs) of Triglycerides, HDL-C, LDL-C, Total Cholesterols, TG/HDL, HbA1c, Fasting Blood Glucose, Insulin, HOMA-IR, and Waist Circumferences according to hsCRP levels in the FiLWHEL study.

	**ORs (95%CIs) according to quintiles of hsCRP levels**					
**(n=371)**	**Q1**	**Q2**	**Q3**	**Q4**	**Q5**	***p-trend***
Triglycerides (≥150 mg/dL) Case/Total number	(4/79)	(5/67)	(10/77)	(8/74)	(12/74)	
Model 1*	1	1.76 (0.45 - 6.94)	2.95 (0.88 - 9.92)	2.54 (0.72 - 8.91)	4.48 (1.34 - 14.94)	0.038
Model 2**	1	1.54 (0.38 - 6.34)	2.67 (0.76 - 9.33)	2.13 (0.59 - 7.71)	4.17 (1.21 - 14.34)	0.061
Model 3***	1	1.43 (0.34 - 6.01)	2.26 (0.63 - 8.11)	1.69 (0.45 - 6.03)	3.33 (0.94 - 11.76)	0.293
High Density Lipoprotein Cholesterol (<40 mg/dL) Case/Total number	(2/79)	(4/67)	(5/77)	(7/74)	(9/74)	
Model 1*	1	2.34 (0.41 - 13.29)	2.68 (0.50 - 14.28)	4.15 (0.83 - 20.83)	5.56 (1.14 - 27.13)	0.153
Model 2**	1	2.35 (0.41 - 13.65)	2.73 (0.50 - 14.83)	4.40 (0.86 - 22.52)	5.62 (1.13 - 28.02)	0.153
Model 3***	1	2.18 (0.37 - 12.88)	2.30 (0.41 - 12.97)	3.61 (0.68 - 19.06)	4.23 (0.82 - 21.84)	0.193
Low Density Lipoprotein Cholesterol (≥130 mg/dL) Case/Total number	(11/79)	(9/67)	(18/77)	(19/74)	(17/74)	
Model 1*	1	1.18 (0.45 - 3.11)	2.03 (0.87 - 4.73)	2.46 (1.06 - 5.71)	2.36 (0.99 - 5.60)	0.033
Model 2**	1	1.07 (0.39 - 2.96)	1.79 (0.73 - 4.37)	1.96 (0.81 - 4.76)	2.10 (0.84 - 5.22)	0.090
Model 3*** (Case=76)	1	1.00 (0.36 - 2.80)	1.61 (0.65 - 4.02)	1.54 (0.62 - 3.86)	1.63 (0.63 - 4.19)	0.024

Total Cholesterol (≥200 mg/dL)						
Case/Total number	(16/79)	(14/67)	(29/77)	(22/74)	(24/74)	
Model 1*	1	1.33 (0.58 - 3.06)	2.70 (1.28 - 5.69)	1.95 (0.90 - 4.19)	2.53 (1.17 - 5.48)	0.003
Model 2**	1	1.31 (0.55 - 3.09)	2.66 (1.23 - 5.77)	1.79 (0.81 - 3.95)	2.45 (1.10 - 5.45)	0.005
Model 3***	1	1.23 (0.51 - 2.93)	2.32 (1.05 - 5.15)	1.45 (0.64 - 3.29)	1.97 (0.86 - 4.51)	0.002
TG/HDL (≥3)						
Case/Total number	(2/79)	(4/67)	(14/77)	(11/74)	(12/74)	
Model 1*	1	2.57 (0.45 - 14.61)	8.97 (1.96 - 41.18)	7.44 (1.58 - 35.14)	8.77 (1.86 - 41.36)	0.000
Model 2**	1	2.18 (0.37 - 12.77)	8.30 (1.77 - 38.81)	6.77 (1.40 - 32.63)	8.02 (1.68 - 38.44)	0.001
Model 3***	1	1.88 (0.31 - 11.50)	7.12 (1.48 - 34.28)	5.21 (1.05 - 25.94)	6.42 (1.30 - 31.61)	0.001
HbA1c (≥5.7%)						
Case/Total number	(27/79)	(16/67)	(27/77)	(27/74)	(33/74)	
Model 1*	1	0.80 (0.37 - 1.71)	1.15 (0.57 - 2.31)	1.28 (0.63 - 2.58)	2.15 (1.06 - 4.34)	0.222
Model 2**	1	0.81 (0.37 - 1.78)	1.09 (0.52 - 2.27)	1.17 (0.57 - 2.40)	1.98 (0.95 - 4.12)	0.350
Model 3***	1	0.74 (0.33 - 1.66)	0.86 (0.40 - 1.85)	0.93 (0.44 - 1.97)	1.53 (0.72 - 3.29)	0.890
Fasting blood glucose (≥100 mg/dL)						
Case/Total number	(8/79)	(6/67)	(8/77)	(13/74)	(8/74)	
Model 1*	1	1.22 (0.39 - 3.86)	1.07 (0.37 - 3.11)	2.28 (0.85 - 6.10)	1.46 (0.50 - 4.27)	0.667
Model 2**	1	1.04 (0.32 - 3.40)	1.00 (0.33 - 3.03)	2.18 (0.79 - 5.98)	1.30 (0.42 - 3.99)	0.716

Model 3***	1	0.98 (0.30 - 3.24)	0.82 (0.26 - 2.55)	1.79 (0.63 - 5.06)	1.02 (0.32 - 3.23)	0.903
Insulin (≥ 15 mg/dL)<br>Case/Total number	(6/79)	(5/67)	(12/77)	(12/74)	(21/74)	
Model 1*	1	0.85 (0.25 - 2.96)	2.14 (0.76 - 6.09)	2.17 (0.76 - 6.18)	4.10 (1.53 - 11.03)	0.019
Model 2**	1	0.83 (0.24 - 2.93)	2.26 (0.78 - 6.50)	2.30 (0.79 - 6.67)	4.47 (1.63 - 12.26)	0.014
Model 3***	1	0.64 (0.17 - 2.40)	1.45 (0.48 - 4.42)	1.45 (0.47 - 4.44)	3.06 (1.06 - 8.87)	0.139
HOMA-IR (≥3.16)<br>Case/Total number	(8/79)	(7/67)	(16/77)	(11/74)	(22/74)	
Model 1*	1	1.02 (0.35 - 2.99)	2.31 (0.92 - 5.78)	1.51 (0.57 – 4.00)	3.57 (1.46 - 8.73)	0.013
Model 2**	1	0.93 (0.31 - 2.78)	2.28 (0.89 - 5.)	1.57 (0.58 - 4.23)	3.61 (1.44 - 9.03)	0.014
Model 3***	1	0.79 (0.25 - 2.47)	1.57 (0.59 - 4.22)	1.00 (0.35 - 2.87)	2.48 (0.94 - 6.52)	0.139
**(n=368)**						
Waist Circumferences (≥80cm)<br>Case/Total number	(25/79)	(23/66)	(37/76)	(36/73)	(43/74)	
Model 1*	1	1.41 (0.69 - 2.91)	2.3 (1.17 - 4.54)	2.55 (1.28 - 5.09)	4.23 (2.09 - 8.58)	0.001
Model 2**	1	1.32 (0.63 - 2.73)	2.27 (1.14 - 4.53)	2.57 (1.27 - 5.16)	4.22 (2.06 - 8.65)	0.002
Model 3***	1	0.93 (0.33 - 2.57)	1.13 (0.44 - 2.95)	1.24 (0.44 - 3.46)	2.58 (0.91 - 7.32)	0.501

Abbreviations : FiLWHEL, Filipino Women's Diet and Health Study; DDII, Data Derived Inflammatory Index; HDL-C, High Density Lipoprotein Cholesterol; LDL-C, Low Density Lipoprotein Cholesterol; HbA1c, Hemoglobin A1c; BMI, Body mass index; TG/HDL, Triglycerides/High density lipoprotein cholesterols; HOMA-IR, Homeostasis Model Assessment of Insulin Resistance.

*Model 1 adjusted for age(years, continuous) and energy intake(kcal, continuous).

**Model 2 additionally adjusted for education level (high school or less, associate/vocational, college or above), sleep hours (<5,5-6,7-8,>8 hours), vigorous activity (yes, no), breastfeeding length (months, continuous), dietary behavior change (yes, no), and alcohol intake (yes, no).

***Model 3 additionally adjusted for BMI (<20, 20-<23, 23-<25, ≥25 kg/m^2).

Supplementary Table 9. Subgroup analyses of the association between DDII, and TG and HOMA-IR in the FiLWHEL study

	TG*			HOMA-IR*		
**(n=405)**	**Q1-3**	**Q4,5**	***p-interaction***	**Q1-3**	**Q4,5**	***p-interaction***
Age, years			0.180			0.807
<35	1	2.05 (0.52 - 8.19)		1	5.48 (2.18 - 13.81)	
Case/Total number	(9/159)	(5/43)		(17/159)	(15/43)	
≥35	1	6.71 (1.78 - 25.33)		1	6.34 (2.28 - 17.66)	
Case/Total number	(3/88)	(22/115)		(6/88)	(27/115)	
Body Mass Index, kg/m^2			0.671			0.969
<25	1	4.13 (1.18 - 14.52)		1	2.60 (0.85 - 7.99)	
Case/Total number	(10/227)	(8/58)		(18/227)	(8/58)	
≥25	1	1.05 (0.15 - 7.20)		1	2.59 (0.68 - 9.86)	
Case/Total number	(2/20)	(19/100)		(5/20)	(34/100)	
Length of residence, years			0.097			0.581
≤8	1	2.24 (0.71 - 7.12)		1	5.35 (2.29 - 12.52)	
Case/Total number	(10/171)	(7/59)		(19/171)	(19/59)	
>8	1	2.23 (54.30 - 8.19)		1	8.84 (2.59 - 30.21)	
Case/Total number	(2/76)	(20/99)		(4/76)	(23/99)	

Abbreviations : DDII, Data Derived Inflammatory Index; FiLWHEL, Filipino Women's Diet and Health Study; TG, Triglycerides, HOMA-IR, Homeostasis Model Assessment of Insulin Resistance; QOL, Quality of life; BMI, Body mass index.

* Adjusted for age(years, continuous). energy intake(kcal, continuous), education level (high school or less, associate/vocational, college or above), sleep hours (<5,5-6,7-8,>8 hours), vigorous activity (yes, no), breastfeeding length (months, continuous), dietary behavior change (yes, no), and alcohol intake (yes, no)

Supplementary Table 10. Odds ratios (ORs) and 95% confidence intervals (CIs) of BMI, Waist Circumferences, Triglycerides, HDL-C, LDL-C, Total Cholesterols, TG/HDL, HbA1c, Fasting Blood Glucose, Insulin and HOMA-IR, according to the MDD-W scoring in the FiLWHEL study.

	**ORs (95%CIs) according to the tertiles of MDD-W**			
	**≤4**	**5**	**≥6**	***p-trend***
Waist Circumferences (≥80cm)				
Case/Total number	(82/176)	(43/96)	(52/139)	
Model 1*	1	0.88 (0.53 - 1.47)	0.60 (0.37 - 0.96)	0.036
Model 2**	1	0.89 (0.53 - 1.50)	0.58 (0.36 - 0.94)	0.029
Model 3†	1	0.68 (0.32 - 1.45)	0.53 (0.27 - 1.05)	0.065
Triglycerides (≥150 mg/dL)				
Case/Total number	(13/177)	(7/96)	(19/132)	
Model 1*	1	0.96 (0.37 - 2.52)	2.04 (0.96 - 4.32)	0.062
Model 2†	1	0.87 (0.31 - 2.43)	2.77 (1.23 - 6.27)	0.016
High Density Lipoprotein Cholesterol (<40 mg/dL)				
Case/Total number	(11/177)	(3/96)	(14/132)	
Model 1*	1	0.49 (0.13 - 1.80)	1.86 (0.81 - 4.27)	0.150
Model 2†	1	0.45 (0.12 - 1.71)	2.33 (0.96 - 5.62)	0.074
Low Density Lipoprotein Cholesterol (≥130 mg/dL)				
Case/Total number	(32/177)	(16/96)	(28/132)	
Model 1*	1	0.87 (0.45 - 1.71)	1.15 (0.64 - 2.04)	0.668
Model 2†	1	0.87 (0.43 - 1.77)	1.37 (0.75 - 2.51)	0.343
Total Cholesterol (≥200 mg/dL)				
Case/Total number	(40/177)	(23/96)	(46/132)	
Model 1*	1	1.05 (0.57 - 1.91)	1.75 (1.04 - 2.92)	0.036
Model 2†	1	1.05 (0.56 - 1.97)	2.07 (1.20 - 3.55)	0.010

TG/HDL (≥3)				
Case/Total number	(13/177)	(8/96)	(22/132)	
Model 1*	1	1.14 (0.45 - 2.86)	2.54 (1.22 - 5.29)	0.012
Model 2†	1	1.15 (0.43 - 3.09)	3.81 (1.68 - 8.62)	0.001
HbA1c (≥ 5.7%)				
Case/Total number	(58/177)	(37/96)	(46/132)	
Model 1*	1	1.27 (0.74 - 2.18)	0.99 (0.60 - 1.63)	0.980
Model 2†	1	1.20 (0.68 - 2.11)	1.08 (0.65 - 1.80)	0.739
Fasting blood glucose (≥100 mg/dL)				
Case/Total number	(19/177)	(11/96)	(13/132)	
Model 1*	1	1.02 (0.45 - 2.31)	0.81 (0.38 - 1.74)	0.602
Model 2†	1	1.01 (0.43 - 2.39)	0.89 (0.40 - 1.96)	0.777
Insulin (≥ 15 mg/dL)				
Case/Total number	(23/177)	(13/96)	(21/132)	
Model 1*	1	1.09 (0.52 - 2.28)	1.38 (0.72 - 2.64)	0.342
Model 2†	1	1.14 (0.51 - 2.58)	2.03 (0.98 - 4.22)	0.063
HOMA-IR (≥3.16)				
Case/Total number	(27/177)	(15/96)	(23/132)	
Model 1*	1	1.04 (0.52 - 2.07)	1.19 (0.64 - 2.19)	0.590
Model 2†	1	1.04 (0.49 - 2.22)	1.62 (0.83 - 3.20)	0.172

Abbreviations : FiLWHEL, Filipino Women's Diet and Health Study; MDD-W, Minimum Dietary Diversity for Women; HDL-C, High Density Lipoprotein Cholesterol; LDL-C, Low Density Lipoprotein Cholesterol; HbA1c, Hemoglobin A1c; BMI, Body mass index; TG/HDL, Triglycerides/High density lipoprotein cholesterols; HOMA-IR, Homeostasis Model Assessment of Insulin Resistance.

*Model 1 adjusted for age(years, continuous) and energy intake(kcal, continuous).

**Model 2 additionally adjusted for dietary behavior changes (yes, no), employment status(yes, no), smoking cohabitant (yes, no), and region (urban, rural).

†Model 2 and Model 3 additionally adjusted for dietary behavior changes (yes, no), employment status(yes, no), smoking cohabitant (yes, no), region (urban, rural), and BMI (kg/m^2, continuous).

Supplementary Table 11. Odds ratios (ORs) and 95% confidence intervals (CIs) of Triglycerides, HDL-C, LDL-C, Total Cholesterols, TG/HDL, HbA1c, Fasting Blood Glucose, Insulin, HOMA-IR, and Waist Circumferences according to DDII in the FiLWHEL study.

	**ORs (95%CIs) according to three categories of DDII**			
**(n=405)**	**Q1-3**<br>247	**Q4**<br>77	**Q5**<br>81	***p-trend***
Triglycerides (≥150 mg/dL)				
Case/Total number	(12/247)	(8/77)	(19/81)	
Model 1*	1	2.21 (0.84 - 5.84)	5.78 (2.44 - 13.70)	<.001
Model 2**	1	2.13 (0.77 - 5.90)	6.44 (2.56 - 16.20)	<.001
Model 3***	1	1.44 (0.47 - 4.36)	2.47 (0.57 - 10.69)	0.242
High Density Lipoprotein Cholesterol (<40 mg/dL)				
Case/Total number	(14/247)	(6/77)	(8/81)	
Model 1*	1	1.67 (0.59 - 4.71)	2.38 (0.86 - 6.53)	0.087
Model 2**	1	1.73 (0.60 - 4.99)	2.32 (0.83 - 6.52)	0.100
Model 3***	1	0.97 (0.30 - 3.09)	0.54 (0.10 - 2.98)	0.561
Low Density Lipoprotein Cholesterol (≥130 mg/dL)				
Case/Total number	(27/247)	(18/77)	(31/81)	
Model 1*	1	2.16 (1.08 - 4.32)	4.14 (2.13 - 8.02)	<.001
Model 2**	1	2.03 (0.98 - 4.19)	3.90 (1.92 - 7.90)	<.001
Model 3***	1	1.93 (0.85 - 4.39)	3.48 (1.11 - 10.95)	0.031
Total Cholesterol (≥200 mg/dL)				
Case/Total number	(43/247)	(26/77)	(40/81)	
Model 1*	1	2.02 (1.10 - 3.68)	3.57 (1.96 - 6.49)	<.001
Model 2**	1	1.95 (1.04 - 3.63)	3.36 (1.81 - 6.24)	<.001
Model 3***	1	1.59 (0.79 - 3.22)	2.11 (0.78 - 5.68)	0.120
TG/HDL (≥3)				
Case/Total number	(15/247)	(10/77)	(18/81)	

Model 1*	1	2.58 (1.07 - 6.24)	5.27 (2.29 - 12.11)	<.001
Model 2**	1	2.86 (1.11 - 7.35)	6.25 (2.53 - 15.41)	<.001
Model 3***	1	1.66 (0.59 - 4.69)	1.71 (0.41 - 7.09)	0.402
HbA1c (≥ 5.7%)				
Case/Total number	(61/247)	(31/77)	(49/81)	
Model 1*	1	1.54 (0.87 - 2.74)	3.19 (1.79 - 5.70)	<.001
Model 2**	1	1.86 (1.02 - 3.39)	3.59 (1.96 - 6.59)	<.001
Model 3***	1	1.52 (0.78 - 2.99)	2.27 (0.89 - 5.80)	0.082
Fasting blood glucose (≥100 mg/dL)				
Case/Total number	(15/247)	(9/77)	(19/81)	
Model 1*	1	1.35 (0.54 - 3.39)	2.71 (1.19 - 6.16)	0.020
Model 2**	1	1.14 (0.43 - 3.01)	2.61 (1.11 - 6.17)	0.033
Model 3***	1	0.69 (0.24 - 2.02)	0.77 (0.19 - 3.20)	0.652
Insulin (≥ 15 mg/dL)				
Case/Total number	(21/247)	(16/77)	(20/81)	
Model 1*	1	5.17 (2.37 - 11.29)	8.70 (3.88 - 19.48)	<.001
Model 2**	1	5.37 (2.38 - 12.09)	9.67 (4.16 - 22.48)	<.001
Model 3***	1	2.30 (0.92 - 5.76)	1.30 (0.33 - 5.13)	0.418
HOMA-IR (≥3.16)				
Case/Total number	(23/247)	(15/77)	(27/81)	
Model 1*	1	3.33 (1.57 - 7.06)	8.38 (4.01 - 17.52)	<.001
Model 2**	1	3.37 (1.53 - 7.42)	9.73 (4.46 - 21.25)	<.001
Model 3***	1	1.60 (0.66 - 3.90)	1.75 (0.50 - 6.14)	0.328
**(n=411)**				
Waist Circumferences (≥80cm)				
Case/Total number	(49/245)	(52/83)	(76/83)	
Model 1*	1	8.26 (4.49 - 15.17)	58.01 (23.13 - 145.52)	<.001
Model 2**	1	9.01 (4.78 - 16.98)	69.04 (26.59 - 179.27)	<.001
Model 3***	1	1.2 (0.53 - 2.73)	0.89 (0.23 - 3.55)	0.903

Abbreviations : FiLWHEL, Filipino Women's Diet and Health Study; DDII, Data Derived Inflammatory Index;

HDL-C, High Density Lipoprotein Cholesterol; LDL-C, Low Density Lipoprotein Cholesterol; HbA1c, Hemoglobin A1c; BMI, Body mass index; TG/HDL, Triglycerides/High density lipoprotein cholesterols; HOMA-IR, Homeostasis Model Assessment of Insulin Resistance.

[*]Model 1 adjusted for age(years, continuous) and energy intake(kcal, continuous), education level (high school or less, associate/vocational, college or above), sleep hours (<5,5-6,7-8,>8 hours), vigorous activity (yes, no), breastfeeding length (months, continuous), dietary behavior change (yes, no), and alcohol intake (yes, no).

[***]Model 3 additionally adjusted for BMI (kg/m^2, continuous).
